# Volatile-mediated inhibition of *Fusarium* wilt in pigeonpea conferred by *Bacillaceae* members isolated from the host plant’s rhizosphere

**DOI:** 10.1007/s11104-025-08093-z

**Published:** 2025-11-26

**Authors:** Rashi Tyagi, Sébastien Bruisson, Laure Weisskopf, Shilpi Sharma

**Affiliations:** 1 Department of Biochemical Engineering and Biotechnology, Indian Institute of Technology Delhi, New Delhi, 110016 India; 2 Department of Biology, University of Fribourg, 1700 Fribourg, Switzerland; 3 Food Research and Innovation Center, University of Fribourg, 1700 Fribourg, Switzerland

**Keywords:** *Bacillaceae*, Antagonism, Vascular wilt, Volatilome, Microcosms

## Abstract

**Background and aim:**

Different biotic and abiotic stresses in agriculture restrict crop productivity, leading to dwindling biomass. Conventional bioinoculants have lost farmers' confidence due to reduced persistence under natural conditions. The understanding of mechanisms underlying suppression of phytopathogens remains a key challenge in developing effective biocontrol strategies. The current study assessed bacterial volatilomes for their ability to inhibit the fungal pathogen *Fusarium udum* in pigeonpea and also to characterize the chemical dialogue underlying the interaction between the pathogen and the biocontrol strains.

**Methods and results:**

The effects of bacterial volatiles on *Fusarium* wilt in pigeonpea investigated through *in planta* assays, applied before and after infection, revealed both direct, antifungal effects, and indirect, plant immunity-mediated effects. The potential dialogue between the biocontrol strains and *F. udum*, studied by monitoring the changes in bacterial volatile emission in strains exposed to the volatiles of the phytopathogenic fungus using connected microcosms, led to the identification of bacterial volatiles whose emission was upregulated upon exposure to *F. udum*. Applying these as synthetic compounds resulted in successful mitigation of *Fusarium* wilt in pigeonpea.

**Conclusion:**

The volatile organic compounds (VOCs) emitted by native members of the pigeonpea (*Cajanus cajan* L. Millsp.) microbiota effectively protected this crop against vascular wilt caused by *F. udum* under controlled conditions. Further, the potential of synthetic volatiles was demonstrated as effective biocontrol strategy, providing a promising alternative to chemical pesticides and inconsistent conventional bioinoculants.

**Supplementary Information:**

The online version contains supplementary material available at https://doi.org/10.1007/s11104-025-08093-z.

## Introduction

Volatile organic compounds (VOCs) mediate mutualistic associations between plants and their associated microbiota (Garbeva and Weisskopf 2020; Russo et al. 2022). These associations can also induce the emission of specific VOCs, highlighting the complex regulation of their synthesis. In recent years, diverse microbial VOCs have been recognized for their ability to promote plant fitness and management of stresses either by inhibiting pests and pathogens and/or inducing plant defense against biotic and abiotic stresses (Bailly and Weisskopf 2017; Brosset and Blande 2022).

In an attempt to find promising biological fumigants against *Botrytis cinerea*, a causal agent of gray mold in grapes, several VOCs emitted by an antagonistic strain of *Pseudomonas fluorescens* were identified by Zhong et al. (2021). Selected volatiles inhibited the fungal mycelia in vitro, suggesting their biocontrol potential. Joo and Hussein (2022) also reported inhibition of the phytopathogens, *Sclerotinia nivalis*, *Cylindrocarpon destructans*, *Botrytis cinerea* and *Alternaria panax,* by VOCs emitted by *Trichoderma* spp. In addition to biocontrol, VOC-mediated growth promotion was also observed in *Raphanus sativus*. Similarly, Awan et al. (2023) investigated the volatiles produced by *Bacillus subtilis*, which exhibited strong antagonism against *Alternaria solani*, the causal agent of early blight in tomato. The extract included various potent antifungal volatiles including octadecane, eicosane, n-tridecan-1-ol, pyrazine-1,4-dione, etc. Reports on the significance of microbial volatiles in combatting biotic stress in plants have recently been reviewed (Garbeva and Weisskopf 2020).

Several abiotic and biotic factors including pH, temperature, nutrient availability, physiological and growth status, and interactions with other microbes affect volatile emission in a microorganism (Insam and Seewald 2010; Rizaludin et al. 2023). Schulz-Bohm et al. (2015) determined the volatile-mediated inter-specific interactions in the rhizosphere by inoculating members of different bacterial genera, including *Paenibacillus*, *Pseudomonas*, *Janthinobacterium*, *Dyella,* and *Burkholderia,* as monocultures and as a synthetic microbial community into soil. The results suggested a substantial effect of *Paenibacillus* on the volatiles released by other member strains of community. Interestingly, trade-offs between tolerance to volatiles emitted by the biocontrol strain *Bacillus amyloliquefaciens* vs. growth and virulence of the pathogen *Ralstonia solanacearum* were reported (Wang et al. 2023). This study highlighted microbial VOCs as both suppressive agents and evolutionary drivers that can select for pathogens with reduced virulence. In response to functionally and structurally different volatile compounds, microorganisms can change their virulence, motility, growth, antibiotic resistance, and synthesis of secondary metabolites, highlighting the significance of studying volatile-mediated interactions between microorganisms (Weisskopf et al. 2021).

Pigeonpea (*Cajanus cajan* L. Millsp.) is a key protein source in African and Asian diets, also exhibiting soil-enriching ability, adaptability, and low fertilizer needs (Ravikumara et al. 2022). Vascular wilt in pigeonpea (caused by *Fusarium udum*) significantly reduces yield, causing up to economic losses of $71 million in India and $5 million in Africa (Pande et al. 2013). The current management practices are not satisfactory in terms of mitigating the disease for a longer time, thus calling for newer approaches. Several reports have emphasized the significant role of volatiles emitted by microbial strains in inhibition of various species of the phytopathogen *Fusarium* (Guevara-Avendaño et al. 2019; Li et al. 2018). The current study aimed to explore the volatile-facilitated biocontrol of the pathogen *F. udum* by indigenous bacterial strains isolated from the rhizosphere of pigeonpea. First, in vivo plant growth experiments investigated the effects of the bacterial strains’ volatilomes on inhibition of *Fusarium* wilt in pigeonpea. Next, changes in the volatilomes of biocontrol strains upon exposure to volatiles of *F. udum* were monitored, and shortlisted volatiles identified as upregulated upon exposure to the pathogen’s volatiles were tested for their biocontrol efficacy against wilt.

## Materials and methods

### Microorganisms

The phytopathogen *Fusarium udum* (NAIMCC-F-02842) was procured from the National Agriculturally Important Microbial Culture Collection (NAIMCC), Indian Council of Agricultural Research-National Bureau of Agriculturally Important Microorganisms (ICAR-NBAIM), Mau Nath Bhanjan, India, and was grown on potato dextrose agar (PDA) at 28 °C. The five selected bacterial strains antagonistic to the phytopathogen *F. udum,* namely *Bacillus haynesii* (DI6; PP683115), *Priestia aryabhattai* (DI15; PP683117), *Rossellomorea marisflavi* (DI18; PP683118), *Bacillus halotolerans* (DC12; PP683122), and *Bacillus stercoris* (Ph7; OQ346092), were isolated from the rhizosphere of pigeonpea grown under different agroclimatic zones in India (codes assigned to bacterial strains and their NCBI accession numbers are given in brackets). The bacterial cultures were cultivated on nutrient agar (NA) plates at 30 °C (Tyagi et al. 2025a).

### *In planta* assay to test the antagonistic activity of volatiles emitted by biocontrol strains against *F. udum*

The seeds of pigeonpea cultivar Bahar were procured from National Seed Corporation Ltd. (NSC), New Delhi, India. The pigeonpea seeds were disinfected by immersing them in 70% ethanol for 40 s, followed by treatment with 0.1% sodium hypochlorite (NaOCl) for 120 s. Subsequently, they were rinsed with autoclaved water to eliminate residual NaOCl (Tewari and Sharma 2020). The surface sterilized seeds were sown in pots (diameter 7 cm) filled with potting soil and grown in a plant growth chamber until the two-leaflet stage. A modified magenta box setup was used to test the potential of volatiles emitted by selected bacterial strains in mitigating *Fusarium* wilt in pigeonpea (Park et al. 2015). The setup was done in two ways to check if the volatiles have antifungal activity or play a role in induced resistance against wilt.

#### Co-inoculation of plants with bacterial volatiles and test pathogen

In this setup, 10 mL of *F. udum* suspension containing 10^6^ spores/mL was drenched near the base of the stem, closer to the roots of pigeonpea seedlings. Then, the potted seedlings were positioned in magenta boxes, each containing a Petri dish at the bottom inoculated with overnight grown bacterial cultures adjusted to an OD_600_ of 1 (five 10 µL aliquots of the suspension were spotted onto small Petri dishes containing NA). A filter and a metal mesh were placed between the Petri dish and the pots to facilitate the transfer of volatiles while providing physical support to the pots.

After 5 days of volatile exposure, the pots were taken out of the boxes and grown at 28 ± 2 °C under a photoperiod of 12 h until the appearance of wilt symptoms in infected control plants. The disease severity score was calculated using a scoring table (Tyagi et al. 2025a). The experiment was performed with six biological replicates, each having three plants.

#### Priming of seedlings with volatiles before pathogen attack

The setup of pigeonpea seedlings in magenta boxes was arranged as described above, with the difference that pathogen inoculation was done after the five initial days of co-incubation of the plants with the bacterial cultures, to avoid any direct contact between the pathogen and the bacterial volatiles. Further incubation outside of the magenta box and disease scoring were carried out as described above. In this second experimental setup (priming), the plants were five days older than in the first one (co-inoculation) when inoculated with the pathogen, however, both experiments contained separate control plants, with which the respective treatments were compared.

### Single microcosm setup for volatiles emitted by individual strains

Before the microcosm setup, the OD_600_ of overnight-grown bacterial cultures was adjusted to 1. Subsequently, five 10 µL drops of the bacterial suspension were spotted onto 5 cm glass Petri dishes filled with NA, and the experiment was set the following day. For *F. udum*, a 5 mm fungal disc was incubated on PDA four days prior to the experiment.

A closed-loop stripping analysis (CLSA) apparatus involving polytetrafluoroethylene (PTFE) microcosms was used to collect volatiles from microbial strains (Hunziker et al. 2015). In the setup, an ACO-388D electromagnetic air compressor (Hailea^R^, China) pumped air at a flow rate of 0.5 mL/min to the microcosm after passing through an air purifying filter. The glass plate with microbial strain was kept in a hermetically sealed microcosm connected to a collection filter with activated charcoal to trap the emitted volatiles. After 48 h, the trapped volatiles were eluted by washing the charcoal filter thrice using 25 µL dichloromethane (DCM). The samples were preserved until analysis at −80 °C. Eight biological replicates were used for each microbial strain, and uninoculated NA and PDA served as controls.

### Dual microcosm setup for assessing volatile-mediated interaction between two microorganisms

A dual microcosm setup was adopted to collect volatiles emitted by biocontrol strains after exposure to the volatiles of the phytopathogen *F. udum* (Bruisson et al. 2023). The glass Petri dishes with *F. udum* and each biocontrol strain were kept in hermetically closed microcosms joined through PTFE tubings. After purification, the airflow from the compressor reached the microcosm harboring *F. udum* and moved to another microcosm with the biocontrol strain, which was connected to a collection filter. Following this unidirectional exposure of volatiles from the test pathogen for 48 h, the trapped volatiles of biocontrol strains were eluted in the same way as described above.

### Gas chromatography/Quadrupole time of flight (GC/Q-TOF) conditions and analysis

The analysis of total volatiles was performed using 7890B GC system connected to a 7250 GC/Q-TOF (Agilent) (Bruisson et al. 2023). The injection of samples was done in an HP-5MS column with a length of 30 m, an inner diameter of 0.25 mm, and a film thickness of 0.25 µm. The parameters were as follows: He flow at 4 mL/min with 2 µL of injection volume and transfer line 300 °C, injector 250 °C, and electron energy of 70 eV. The program featured 5 min at 50 °C followed by an increase of 5 °C/min up to 320 °C and 1 min hold. The acquisition of mass spectra was done in centroid mode (20–400 m/z, 3 scans/s). An alkane retention standard solution injected under the same conditions was used to calculate the retention index (RI).

All GC/Q-TOF data were processed using MzMine 3.9 after converting the raw ".D" files into the ".mzdata" format (Schmid et al. 2023). Mass detection was conducted before chromatogram construction and deconvolution using the Automated Data Analysis Pipeline (ADAP) algorithm (Du et al. 2020). Spectral deconvolution was subsequently carried out using the multivariate curve resolution method. Feature alignment was performed using the Join aligner, followed by statistical analysis on the MetaboAnalyst 6.0 online platform (Pang et al. 2024). Volatile compounds were identified using NIST MS Search v.2.3 software.

### *In planta* assay to check the inhibition of *F. udum* by selected synthetic volatile compounds

Three surface sterilized pigeonpea seeds were transferred to plastic cups filled with soil, which had small holes at the bottom. After the germination of seedlings, these cups were fixed in another cup containing an inverted lid of a centrifuge tube containing 100 mg of selected synthetic volatiles. The cups were sealed with parafilm to prevent the escape of volatiles. Next, at the two-leaflet stage, a spore suspension of *F. udum* (10^6^ spores/mL) was drenched at the base of stem, closer to the roots of seedlings. This plastic cup setup was incubated in a growth chamber at 28 ± 2 °C temperature, 70–80% humidity, and 12 h photoperiod. The plants with no exposure to volatiles or pathogen inoculation served as uninfected control, while plants drenched with *F. udum* but not exposed to volatiles served as infected control. Every treatment had seven biological replicates, each with two plants (Tahir et al. 2017). Phenotypic parameters like disease severity index, shoot length, root length, biomass of seedlings, and total chlorophyll content were documented once wilt symptoms appeared in infected control plants. Further, lipid peroxidation (LPX), proline content, and total phenolics were also analysed using standard procedures (Bates et al. 1973; Heath and Packer 1968; Mallick and Singh 1980). Additionally, activities of antioxidant enzymes, including superoxide dismutase (SOD, EC 1.15.1.1) (Beauchamp and Fridovich 1971), ascorbate peroxidase (APX, EC 1.11.1.11) (Mishra et al. 2006), glutathione peroxidase (GPX, EC 1.11.1.7) (Hemeda and Klein 1990), phenylalanine ammonia lyase (PAL, EC 4.3.1.24), and polyphenol oxidase (PPO, EC 1.14.18.1) (Lavania et al. 2006), were also determined.

### Statistical analysis

The data on volatile compounds was filtered, normalized, and scaled using Metaboanalyst. Further, one-way analysis of variance (ANOVA) followed by Fisher's least significant difference (LSD) post hoc test (p < 0.05) was performed to generate a list of significantly different compounds produced between fungal strain, unexposed bacterial strain, and bacterial strain when exposed to the fungal strain, in a comparison group. The principal component analysis and hierarchical clustering were done using prcomp package and Euclidean distance and ward.D clustering algorithm, respectively.

For plant growth experiments involving volatiles, treatment was considered as the independent and disease severity/stress marker as the dependent variable and one-way ANOVA was done through SPSS Statistics 16.0 (SPSS Inc., Chicago, Ill., USA) with Duncan alpha post-hoc test at p < 0.05.

## Results

### Disease suppression ability of bacterial volatilomes *in planta*

**Fig. 1:**
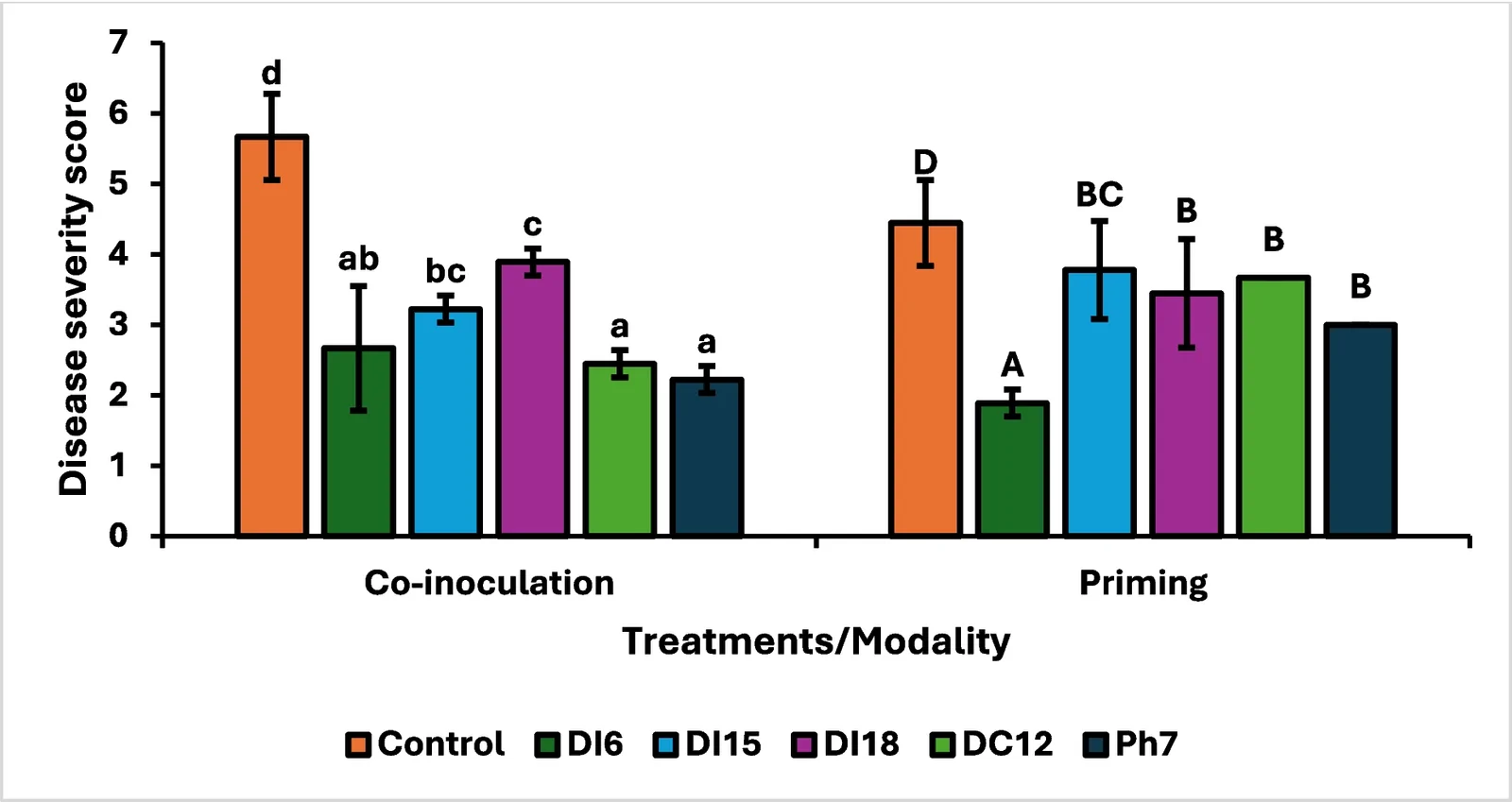
*In planta* biocontrol activity of volatiles released by bacterial strains under two treatment modalities, co-inoculation and priming. The plot shows the disease severity score observed in plants treated with volatilome of bacterial strains (differentiated by colours). Treatments are compared with their respective controls within each modality using one-way ANOVA. Different lowercase letters indicate statistically significant differences among co-inoculation treatments (p < 0.05), while uppercase letters indicate significant differences among priming treatments (p < 0.05) based on Duncan post-hoc test. Error bars represent standard deviation where *n* = 6. The experiment was performed with six biological replicates, each having three plants and each plant was scored separately

To check if the volatilomes (i.e. the complex mixture of volatile compounds) released by bacterial strains antagonistic to *F. udum* would protect pigeonpea against vascular wilt, an *in planta* assay was set up. The *F. udum* infected plants not exposed to bacterial volatilomes displayed highest wilt severity, with scores in the range of 4.4 to 5.6 (Fig. 1).

All the treatments with bacterial volatilomes for both the modalities, co-inoculation and priming, reduced the severity of wilt in host plants as compared to their respective *F. udum* infected control. For co-inoculation, the highest disease protection was observed in plants treated with the volatilome of *B. stercoris* (Ph7) and *B. halotolerans* (DC12). In contrast, when plants were primed with the bacterial volatilomes prior to infection, the best performing strain was *B. haynesii* (DI6), which led to the lowest disease score of 1.8, less than half of that observed in the *F. udum* infected control.

### Exposing the biocontrol strains to the volatilome of *F. udum* led to higher emission of several volatiles

**Fig. 2:**
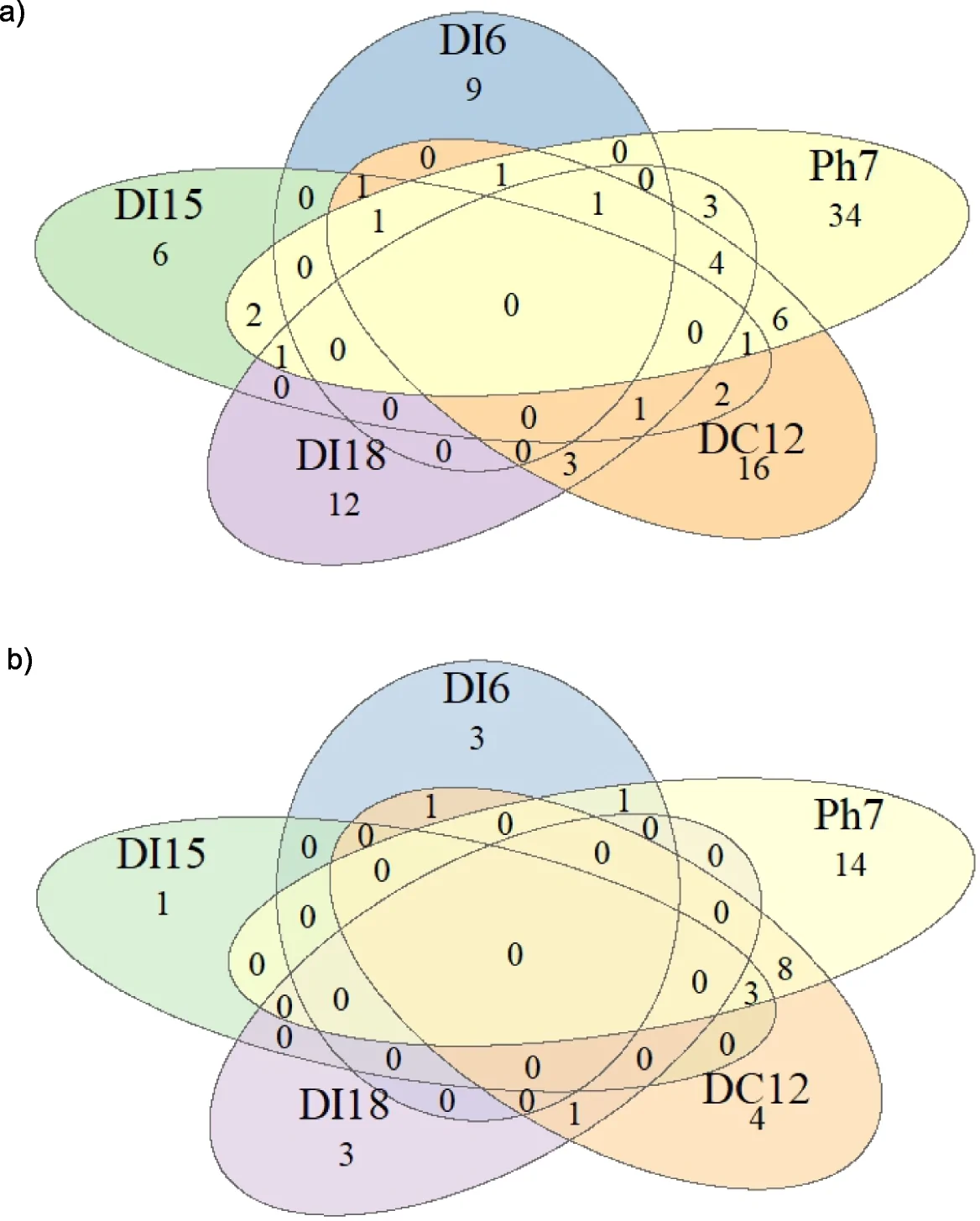
Venn diagrams showing the distribution of the volatile organic compounds (VOCs) emitted by five biocontrol strains, (**a**) VOCs produced by the strains when grown individually in a single microcosm setup, and (**b**) VOCs emitted in higher amounts by the strains when exposed to volatiles released by the test pathogen, *F. udum*. The numeric values represent the compounds unique to individual strains as well as those shared among multiple strains. Each strain is represented by a different colour

Since for most strains, higher efficacy was observed in the plant protection assay in the co-inoculation setup than in the priming setup, the next step was to check whether the presence of the pathogen would induce the emission of particular volatile compounds. The volatilome profiles of the five biocontrol strains were first analysed when strains grew individually in a single microcosm setup. This revealed different profiles for strains even when belonging to the same family, *Bacillaceae*, while we also observed few shared VOCs between strains, especially *B. stercoris* (Ph7) and *B. halotolerans* (DC12) (Fig. 2a, Table S1). 1-Tridecene and oxacyclododecane-2-one were identified as the most common compounds, emitted by four out of five strains. Additionally, several ketones such as 2-dodecanone, 2-nonanone, 2-tridecanone, flopropione, were commonly produced by at least three of the strains.

When comparing the volatilomes obtained upon exposure of the bacterial strains to the volatiles of *Fusarium udum*, we observed differential emission of several volatiles. The principal component analysis (PCA) showed clear demarcations between exposed and unexposed biocontrol strains (Fig. S1a), which was illustrated through clustering analysis and heatmaps (Fig. S1b). The heatmaps highlighted only the significantly different mass features between groups, revealing three distinct clusters corresponding to the three groups.

Each biocontrol strain exhibited a unique response to exposure of the fungal volatiles, with some strains showing a greater number of modifications in their volatilome, while others had fewer changes. Figure 2b summarizes the number of common and unique volatiles that were upregulated when exposed to volatiles of *F. udum*.

A list of volatile compounds upregulated in the presence of volatiles emitted by *F. udum* and their reported bioactivity is presented in Table 1. Many of these volatile compounds, including butyl sorbate, retinal, and terpinen-4-ol, were common and emitted by more than one strain, while some compounds like tetradecane, linalool, undecanal, were specific to some strains.

**Table 1:** List of volatiles released by bacterial strains with increased abundance when exposed to volatiles from *F. udum*

S. No	Volatile organic compound (VOC)	Mass over charge ratio (m/z)	Kovats retention index (RI)	CAS registry number	Emitter strain/s					Reported biological activity	Reference
					DI6	DI15	DI18	DC12	Ph7		
1	1H-Pyrazole, 1,3,5-trimethyl-	43.0189	1041	1072–91-9		**√**		**√**	**√**	-	
2	Butyl sorbate	112.0914	1261	7367–78-4		**√**		**√**	**√**	-	
3	Cyclohexanemethanol, 4-hydroxy-α,α,4-trimethyl-	96.0960	1227	80–53-5		**√**		**√**	**√**	Broad antifungal activity against molds	Salem et al. 2016
4	2-Methoxy-5-methylphenol	123.0831	1114	1195–09-1				**√**	**√**	-	
5	3-Buten-2-one, 4-(2,2,6-trimethyl-7-oxabicyclo[4.1.0]hept-1-yl)-	123.0839	1269	23,267–57-4				**√**	**√**	-	
6	3-Buten-2-one, 4-(2,2,6-trimethyl-7-oxabicyclo[4.1.0]hept-1-yl)-^1^	123.0839	1147					**√**	**√**		
7	Terpinen-4-ol	111.0832	1085	562–74-3				**√**	**√**	Antifungal activity against *Botrytis cinerea*, *Aspergillus flavus* and mycotoxigenic fungi	Morcia et al. 2012; Ren et al. 2024; Yu et al. 2024
8	Terpinen-4-ol^1^	111.0832	1178					**√**	**√**		
9	Ethanone, 1-(2-methyl-1-cyclopenten-1-yl)-	109.0678	1010	3168–90-9				**√**	**√**	-	
10	1,3-Benzenediol, 4-ethyl-	123.0474	1092	2896–60-8				**√**	**√**	-	
11	Ethanone, 1-cyclopropyl-	69.0718	1017	765–43-5				**√**	**√**	Broad antibacterial and antioxidant activity	Sari et al. 2024
12	4-Methoxycinnamaldehyde	161.1360	1431	24,680–50-0	**√**				**√**	Inhibition of *Penicillium digitatum*	Gan et al. 2020
13	Benzoic acid, 2,6-dichloro-	173.0847	1635	50–30-6			**√**	**√**		-	
14	Naphthalene, 1-isocyano-	153.0938	1554	1984–04–9	**√**			**√**		-	
15	Tetradecane	57.0709	1398	629–59-4	**√**					Plant growth promotion, induced systemic resistance (ISR) in *Vigna radiata*; Antifungal activity against *Rhizopus stolonifer*, *Rhizoctonia solani,* and *Thielaviopsis ethacetica*	Gajera et al. 2020; Jishma et al. 2017
16	9-Hexadecen-1-ol, (Z)-	165.0936	1470	10,378–01–5	**√**					-	
17	Tridecane	71.0508	1350	629–50-5	**√**					Antifungal activity against *Aspergillus* sp, *Thielaviopsis ethacetica,* and *Meloidogyne incognita*; Elicit plant defense gene expression	Boukaew and Prasertsan 2020; Lee et al. 2012; Li et al. 2019
18	Butyl sorbate^1^	112.0911	1298	7367–78-4					**√**	-	
19	9-Octadecen-1-ol, (Z)- (commonly known as oleyl alcohol)	43.0190	1298	143–28-2		**√**				Inhibitory effects of *Fusarium moniliforme* and *Cladosporium cucumerinum*	Gata-Gonçalves et al. 2003; Wang et al. 2013
20	9-Octadecen-1-ol, (Z)-^1^	96.0593	1236						**√**		
21	9-Octadecen-1-ol, (Z)-^2^	95.0881	1170						**√**		
22	Acetic acid, diethyl-	43.0190	938	88–09-5			**√**			Promotion of biomass in *Arabidopsis thaliana*	He et al. 2023
23	Undecanal	56.0633	1004	112–44-7			**√**			Enhancement of root and shoot length in *Arabidopsis thaliana*; Antifungal activity against *Fusarium oxysporum*, *Verticillium dahliae*, and *Phytophthora infestans*	Li et al. 2020; Parmagnani et al. 2023; Rani et al. 2023
24	Retinal	161.1363	1480	116–31-4			**√**			-	
25	Retinal^1^	95.0876	1596						**√**		
26	Retinal^2^	173.084	1635						**√**		
27	3-Buten-2-one, 4-(2,2,6-trimethyl-7-oxabicyclo[4.1.0]hept-1-yl)-^2^	112.0912	1298	23,267–57-4				**√**			
28	3-Buten-2-one, 4-(2,2,6-trimethyl-7-oxabicyclo[4.1.0]hept-1-yl)-^3^	109.0677	1037						**√**		
29	Mercury, bromomethyl-	73.0845	1673	7789–47-1				**√**		-	
30	Linalool	121.1044	1136	78–70-6				**√**		Mitigation of *Fusarium* wilt in tomato; Augmentation of roots in *Brassica rapa*; Inhibition of *Colletotrichum*, *Sclerotinia* and *Fusarium oxysporum*	Li et al. 2023; Moisan et al. 2021
31	2-Hexanone, 3,4-dimethyl-	43.0190	1073	19,550–10-8				**√**		-	
32	Eicosane	73.0484	1673	112–95-8					**√**	Antifungal activity against *Rhizoctonia solani* and other phytopathogenic fungi	Ahsan et al. 2017; Bhat et al. 2024
33	2-Octanone	58.0428	967	111–13-7					**√**	Broad antifungal activity	Inayati et al. 2019
34	?,?-Dichloromethyl methyl ether	81.0018	1065	4885–02–3					**√**	-	
35	Octadecane, 1-isocyanato-	99.0464	1176	112–96-9					**√**	-	
36	Octadecane, 1-isocyanato-^1^	99.0157	1019						**√**		
37	2-Pentanone, 4-hydroxy-4-methyl-	59.0503	71	123–42-2					**√**	Antifungal activity against *Monosporascus cannonballus*	Al-Daghari et al. 2020
38	2-Decanone	58.0426	1164	693–54-9					**√**	Inhibitory activity against several phytopathogen *Aspergillus flavus*, *Aspergillus parasiticus*, *Fusarium solani*, *Rhizopus stolonifer*, *Fusarium oxysporum cubense,* and *Colletotrichum gloeosporioides*; Plant growth promotion in potato and maize	Boukaew and Prasertsan 2020; Guevara-Avendaño et al. 2019; Mülner et al. 2020; Yuan et al. 2012; Zheng et al. 2013
39	Benzyl 2-chloroethyl sulfone	91.0563	1246	66,998–67-2					**√**	-	

^1, 2, 3^ denotes features having same identification regardless of differences in retention index. “**√**” represents that the volatile compound is produced by the bacterial strain listed in that column

### Bioactivity of synthetic volatile compounds *in planta*

**Fig. 3:**
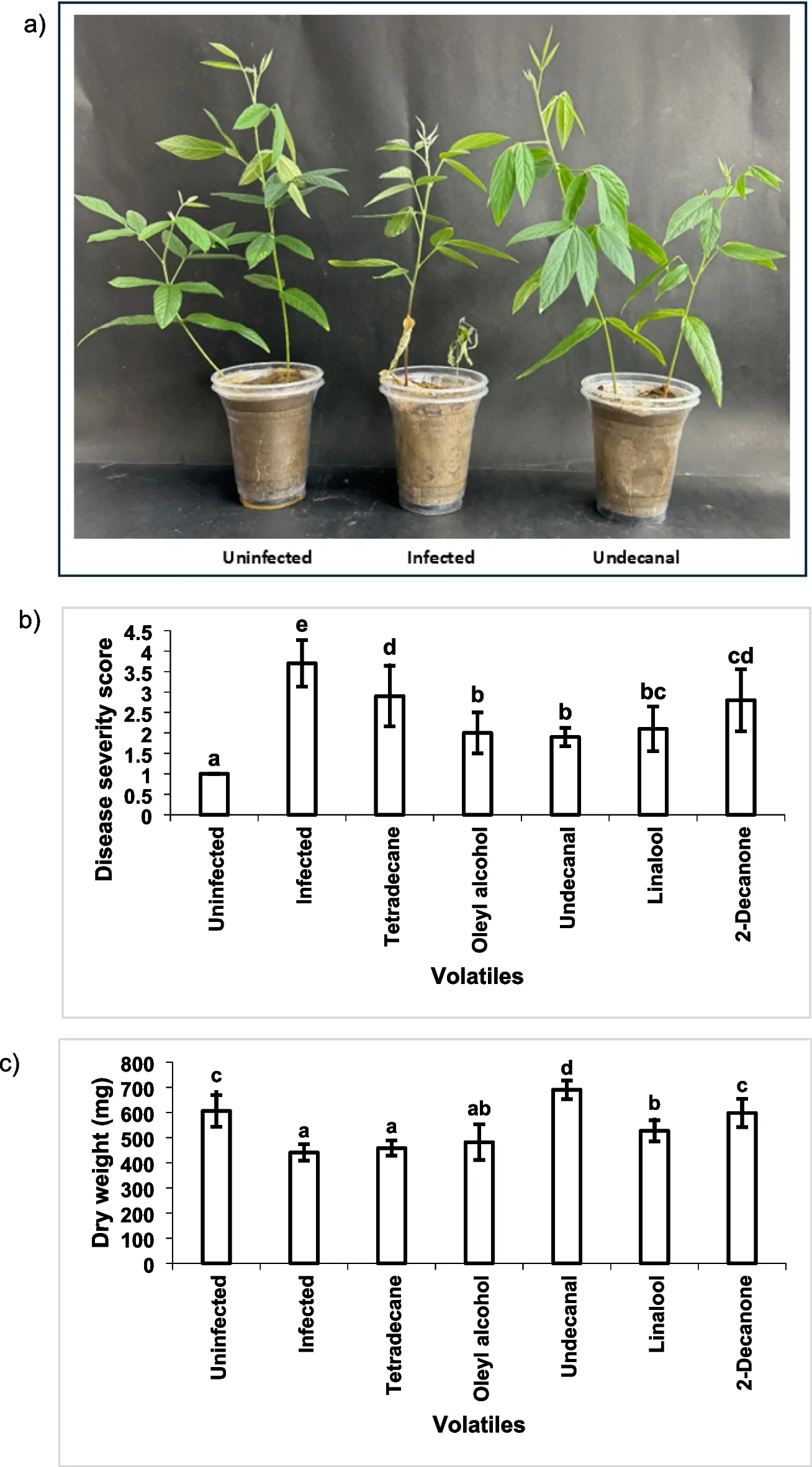
*In planta* study for the suppression of *Fusarium* wilt by pure synthetic volatiles, **a**) Representative pictures showing plants in the order, uninfected, infected, exposed to undecanal (left to right); **b**) Disease severity scores observed in plants exposed to different volatiles; **c**) Dry weight of plants exposed to different volatiles. Error bars represent standard deviation where *n* = 7; statistical differences were analysed through one-way ANOVA. Different letters represent statistical differences (p < 0.05) based on Duncan post-hoc test

From the list of compounds upregulated in response to exposure to the test pathogen, five compounds namely tetradecane, 9-octadecen-1-ol, (Z) (oleyl alcohol), undecanal, linalool and 2-decanone, were selected based on their documented ability to inhibit various *Fusarium* species across different crops, as well as their availability in synthetic form. Their disease protection ability was tested through an *in planta* assay (Fig. 3a and b). The highest incidence of wilt was observed in infected controls (*F. udum* infected plants with no exposure to volatiles), which were assigned the maximum disease severity score of 3.7.

The plants exposed to each of the five volatiles showed a reduced incidence of *Fusarium* wilt in pigeonpea under controlled conditions when compared to the infected controls. Among these five volatiles, three (oleyl alcohol, undecanal, linalool) exhibited the greatest reduction in wilt severity. However, there was no significant difference in the reduction among these three.

The biomass of *F. udum*-infected plants was severely impacted, but it improved considerably when exposed to the three synthetic volatile compounds (Fig. 3c). Undecanal showed the maximum increase of 1.5-fold in plant dry weight, closely followed by 2-decanone and linalool by 1.3 and 1.1-fold, respectively. In contrast, exposure to tetradecane did not induce any significant change in dry weight. For root length, shoot length, and total chlorophyll content, there were no significant differences among the treatments except uninfected and infected controls (data not shown).

### Effect of synthetic volatiles on stress markers and antioxidant enzymes

**Fig. 4:**
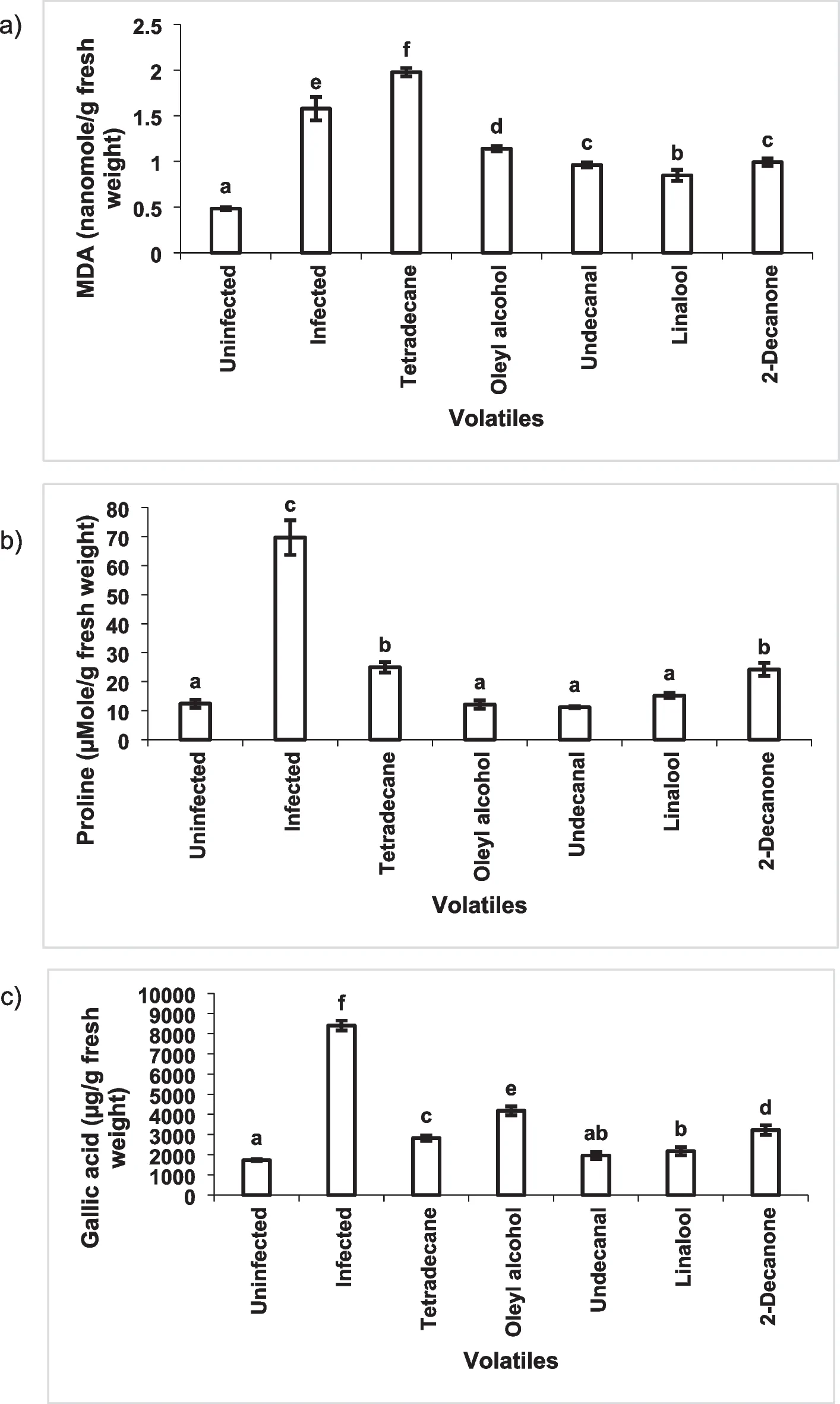
Non-enzymatic stress markers exhibited by plants treated with different synthetic volatiles, **a**) Lipid peroxidation, measured by quantifying malondialdehyde (MDA), **b**) Proline, and **c**) Total phenolics (quantified using gallic acid as reference standard). Error bars represent standard deviation where *n* = 3 (test was conducted in triplicates using a composite sample of seven biological replicates). Statistical differences were analysed through one-way ANOVA. Different letters represent statistical differences (p < 0.05) based on Duncan post-hoc test

A high level of malondialdehyde (MDA) observed in plants treated solely with *F. udum* indicated increased peroxidation of lipids induced by the fungal pathogen (Fig. 4a). However, this MDA content was significantly lowered when plants were exposed to all synthetic volatiles except tetradecane, indicating lesser lipid peroxidation due to limited spread of wilt in pigeonpea plants.

A similar trend was observed for proline, although here all volatiles including tetradecane induced significant decrease in proline levels compared with the high levels observed in the infected controls (Fig. 4b). Exposure to the three volatiles, oleyl alcohol, undecanal, and linalool, even resulted in proline levels comparable to the uninfected control, indicating minimal or no stress caused by the pathogen. Besides proline, a decline in total phenolic content, which was quantified using gallic acid as reference standard, was also noted for all synthetic volatiles in comparison to the infected controls, with the strongest effect for undecanal and linalool (Fig. 4c). In conclusion, linalool and undecanal outperformed the other three volatiles based on the assessed non-enzymatic stress markers.

**Fig. 5:**
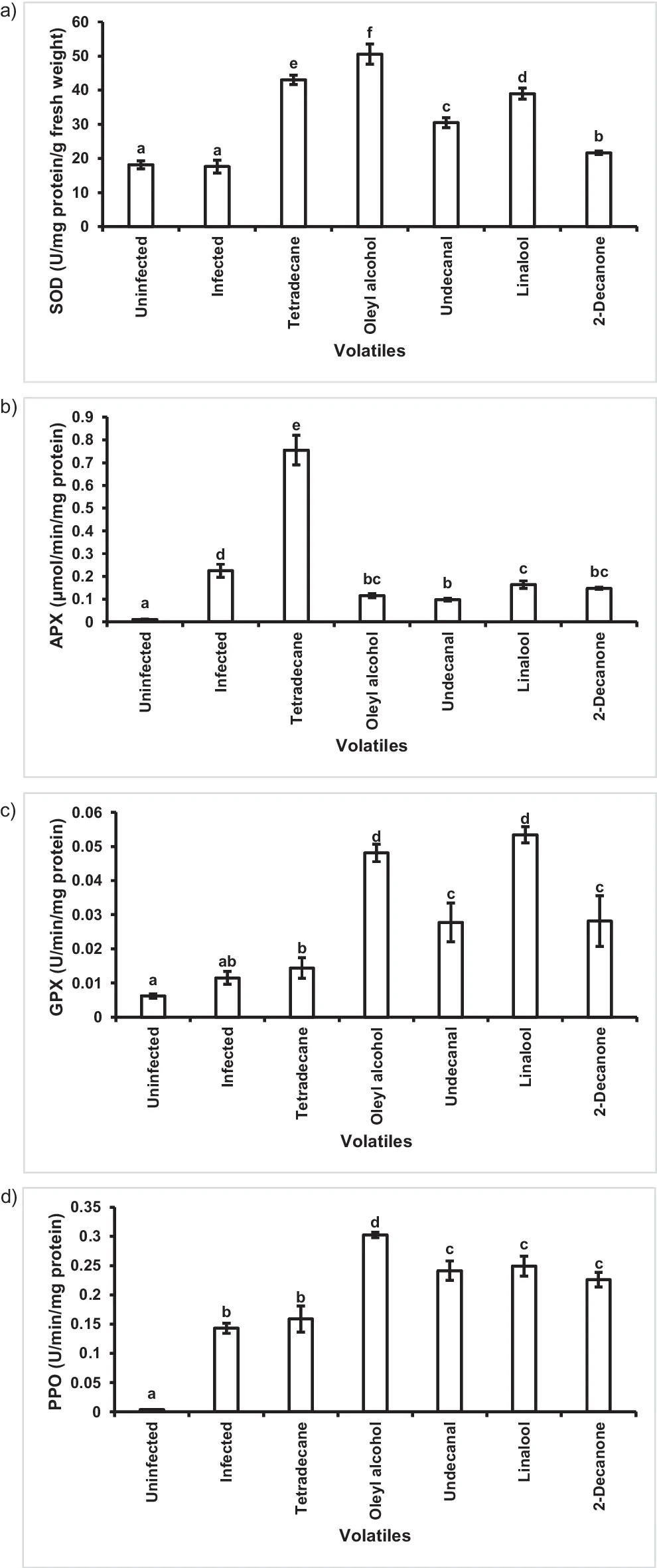
Antioxidant activities shown by plants subjected to various synthetic volatiles, **a**) SOD, **b**) APX, **c**) GPX, and **d**) PPO. Error bars represent standard deviation where *n* = 3 (test was conducted in triplicates using a composite sample of seven biological replicates). Statistical differences were analysed through one-way ANOVA. The same letters express no significant difference (p < 0.05) based on Duncan post-hoc test

With respect to antioxidant enzymes, plants exposed to different volatiles exhibited varying responses for the different antioxidant enzymes tested. For SOD, the enzymatic activity for both uninfected and infected controls remained comparable, while for all five volatiles, there was a significant enhancement in the activity (Fig. 5a). In comparison to the infected control, the highest activity was seen for oleyl alcohol, while the lowest increase occurred with 2-decanone.

Contrarily, the greatest increase in activity of APX was seen upon exposure to tetradecane, while the four other volatiles lowered APX activity compared to the infected control (Fig. 5b). Next, while infection did not result in any increase in GPX activity, elevated activities were reported with all volatiles except tetradecane (Fig. 5c), with the greatest spike occurring in plants exposed to oleyl alcohol and linalool. Similar to APX, the enzymatic activity for PPO was also increased in infected controls in comparison to uninfected controls (Fig. 5d). The activity of this enzyme was further increased upon exposure to oleyl alcohol, and to a lesser extent upon exposure to linalool, undecanal and 2-decanone, while tetradecane exposure did not result in any change compared with the infected control plants. Taking together the results obtained on disease incidence, growth attributes, and non-enzymatic stress markers, all volatiles except tetradecane exhibited a significant contribution in managing vascular wilt in pigeonpea, with linalool, oleyl alcohol, and undecanal emerging out as the best performers.

## Discussion

The rapid increase in world population calls for an increase in crop productivity. However, biotic stresses due to microbial pathogens and pests threaten global agriculture, causing severe crop damage and yield losses (Pradhan et al. 2022). Fungal pathogens are responsible for a 20% reduction in global crop yields, a loss that could otherwise provide food for 600 million people annually (Davies et al. 2021). Additionally, global climate change is anticipated to raise the occurrence of pathogen outbreaks, exacerbating food insecurity. To address this challenge and boost agricultural production, chemical pesticides have become a widely used solution. However, the calamitous after-effects of these agrochemicals on humans and the ecosystem demands a shift towards more sustainable and environment-friendly agro-practices backed by the 2030 agenda of sustainable development (Arif et al. 2020; Hakim et al. 2021). Microbes have been used in agriculture for decades to enhance growth and combat diseases. These biocontrol agents serve as an economical and eco-friendly approach but have limited success owing to inconsistent performance in field conditions, partly due to competition from native soil microflora.

The presence of root exudates in rhizospheric soil aids the recruitment and nurturing of a diverse and abundant microbial flora (Hartmann et al. 2019). This microbiota, thriving in the rhizosphere, is beneficial as it promotes plant growth either directly through biological nitrogen fixation, phytohormone production, nutrient sequestration, etc., or indirectly via competition or synthesis of antimicrobial metabolites, lytic enzymes, etc. Further, the soil microbiota also produces several VOCs known to play an important role in disease suppression through inhibition of plant pathogens and stimulation of plant defenses (Bailly and Weisskopf 2017).

The genus *Fusarium* contains many pathogenic species that are responsible for a variety of diseases in important food crops worldwide (Reddy et al. 2024). One such species, *F. udum,* causes vascular wilt in the staple crop pigeonpea (*Cajanus cajan*) leading to 15–30% of plant mortality. Widespread across Asia, Africa, and North America, this fungal disease is reportedly increasing at an alarming rate, with a significant drop in pigeonpea yields anticipated. The current disease management strategies include systemic fungicides, resistant plant varieties, and conventional biocontrol agents. However, fungicide use poses ecological concerns, while commercially available resistant cultivars exhibit varying susceptibility across different regions due to the high pathogenic variability of the pathogen. Additionally, conventional bioinoculants developed till date have proven ineffective over time, leaving no reliable strategy for the efficient control of the disease (Dukare and Paul 2021; Ravikumara et al. 2022).

Earlier, we characterized the pathobiome linked to *Fusarium* wilt by identifying various bacterial and fungal genera uniquely associated with rhizospheric samples collected from *Fusarium*-infested pigeonpea fields across different agroclimatic zones (Tyagi et al. 2025a). Subsequently, we established a culture bank focusing on indigenous *Bacillus* strains with antagonistic potential against *F. udum*, guided by evidence of negative interactions between *Fusarium* and *Bacillus*, identified through correlation analysis between microbial communities in rhizospheric soil by employing amplicon sequencing. Further, robust synthetic microbial communities were also constituted using these antagonistic strains employing a novel approach of iterative deconvolution, to combat vascular wilt in pigeonpea (Tyagi et al. 2025b).

In a heterogenous system like a soil matrix, the movement of volatile compounds through gaseous phase holds importance (Garbeva and Weisskopf 2020). Within this setting, volatile signals can accumulate and disperse through air-filled soil pores, facilitating long-distance interactions between roots and microbes—beyond the range achievable by diffusible compounds alone. Thus, taking a step forward into constituting some resilient strategies to combat *Fusarium* wilt of pigeonpea using these *Bacillus* strains through rhizospheric engineering, the present study aimed at assessing the ability of bacterial volatilomes to inhibit the fungal pathogen *F. udum* in pigeonpea, and also to better understand the chemical dialogue underlying the interaction between the pathogen and the biocontrol strains.

Bacterial volatiles can act via numerous mechanisms, by directly inhibiting phytopathogens or by triggering induced systemic resistance in plants, or both (Kai et al. 2007; Lee et al. 2012; Li et al. 2023). Sharifi and Ryu (2016) emphasized on the primary role of bacterial volatiles being elicitor of induced systemic resistance in plants. Contrastingly, in the present study, different bacterial strains behaved in a varied manner in the two different setups we tested, i.e. concomitant exposure of pigeonpea plants to bacterial volatilomes and infection with *F. udum,* and priming of plants with bacterial volatilomes before inoculation of the pathogen. However, as observed in plant growth experiments, the overall lesser efficiency of priming compared with co-inoculation (direct contact between bacterial volatilomes and *F. udum*) hinted towards a potential dialogue between the two organisms that may have led to *Fusarium*-induced volatile emission in biocontrol strains.

The adoption of a dual microcosm setup (Bruisson et al. 2023) with a unidirectional exposure of biocontrol strains to the volatiles emitted by *F. udum* allowed the well-defined characterization of the effect of the pathogen’s volatilome on the biocontrol strains’, with no interference of the bacteria on the fungal volatilome, which would not be possible with setups growing both organisms in the same headspace. The results conveyed that all the five biocontrol strains modified their own volatilome composition in the presence of volatiles emitted by *F. udum*. Though each strain exhibited a distinct response to the fungal volatiles, some of the upregulated volatiles were shared by two or three strains, while most were unique to one particular strain. This observation shows both conserved and strain-specific metabolic responses in biocontrol strategies employed by these *Bacillaceae* strains. The presence of commonly upregulated volatiles across strains suggests a shared mechanism for biotic stress mitigation, while the production of unique volatiles by individual strains underscores their specialized biocontrol capacities and ecological differentiation. This is also in line with previous results observed with the same setup on the fungi, *Fusarium oxysporum, Botrytis cinerea,* and *Trichoderma harzianum,* all of which responded differently to the same volatilome exposure, inducing both up- and down-regulation of specific volatiles (Bruisson et al. 2023). Beyond changes in abundance, some compounds may appear exclusively or, conversely, disappear entirely as a result of the interaction between different microbial strains (Rybakova et al. 2022). However, in our study, the primary response of the five biocontrol strains to the perception of fungal volatiles, was an increased abundance of most volatile compounds, with no observed decrease or disappearance, which could indicate a broad-spectrum stress response where the bacteria enhance the production of multiple volatiles as a defense mechanism, the activation of global regulatory pathways (like quorum sensing) that simultaneously activate entire biosynthetic clusters, or the accumulation of antifungal volatiles over time.

Interestingly, out of a total of 39 volatile compounds detected as upregulated upon exposure to the volatiles of *F. udum*, 13 have been reported for their antimicrobial activity against several fungal phytopathogens including *Fusarium*, *Rhizopus*, *Aspergillus*, *Phytophthora,* etc. The increased emission of these antifungal volatiles by the five biocontrol strains might indicate that they play a role in the antagonistic activity against *F. udum*. The volatile-mediated bacterial antagonism of fungal pathogens has already been reported for many strains and many pathogens (Bailly and Weisskopf 2017; Hunziker et al. 2015; Sdiri et al. 2022; Tahir et al. 2017). However, in most studies, volatilome profiling of biocontrol strains has been done in the absence of the pathogen, although the compounds found in higher relative abundance after exposure to the pathogen’s volatiles provide a more targeted and tailored list of compounds likely to exhibit interesting biological activities against the tested pathogen, which also partially overcomes the limitation of pure culture setups wherein induced antimicrobial compounds are overlooked.

Then, taking a step towards next generation bioformulations, and to check the applicability of volatile compounds to eradicate wilt disease in pigeonpea, we selected five upregulated volatiles produced by the biocontrol strains in the current study, based on earlier reports indicating their antagonism against various *Fusarium* sp. and their availability in pure form. These volatiles included tetradecane, undecanal, oleyl alcohol, linalool, and 2-decanone, and we checked their ability to control *Fusarium* wilt in pigeonpea plants. Successful mitigation of *Fusarium* wilt in tomato, augmentation of roots in *Brassica rapa*, and inhibition of *Colletotrichum*, *Sclerotinia*, and *Fusarium oxysporum* through exposure to the volatile linalool have already been reported (Li et al. 2023; Moisan et al. 2021). Similarly, undecanal is also known for its role in enhancement of root and shoot length in *Arabidopsis thaliana* and antifungal activity against *Fusarium oxysporum*, *Verticillium dahliae* and *Phytophthora infestans* (Parmagnani et al. 2023; Rani et al. 2023). Also, the inhibitory activity of 2-decanone against several phytopathogens including *Aspergillus flavus*, *Aspergillus parasiticus*, *Fusarium solani*, *Rhizopus stolonifer*, *Fusarium oxysporum cubense,* and *Colletotrichum gloeosporioides,* and plant growth promotion in potato and maize has been reported (Boukaew and Prasertsan 2020; Guevara-Avendaño et al. 2019; Mülner et al. 2020). In line with these previous reports, most synthetic volatiles tested in our study, including undecanal, oleyl alcohol, linalool, and, to a lesser extent, 2-decanone, led to protection against *F. udum-*induced wilt in pigeonpea plants. Beyond reduced disease incidence, the biomass of plants exposed to undecanal was even higher than uninfected control plants, advocating for its plant growth promoting ability. Though most volatiles are known for their beneficial role in plant fitness, some are phytotoxic such as dimethyl disulphide (Kai 2020). There is also evidence of strong phytotoxicity associated with certain inorganic bacterial volatiles, such as ammonia and hydrogen cyanide (Blom et al. 2011). This phytotoxic potential has led to interest in using such VOC-producing bacteria for the biological control of weeds. Additionally, some organic bacterial volatiles have been reported to exhibit phytotoxic effects albeit only at higher concentrations (Tyagi et al. 2018). In the same way, the wilt incidence and high MDA content in plants exposed to tetradecane might be due to its phytotoxicity in higher concentrations.

The increase in MDA content in plants is associated with lipid peroxidation in the cell membrane, triggered by reactive oxygen species (ROS) in response to biotic stress. This elevated ROS production is influenced by proline metabolism, which affects signaling pathways. Therefore, in addition to serving as an osmolyte, proline functions as both a signaling molecule and an antioxidant defense agent (Kumari et al. 2017; Liang et al. 2013). The noticeable decrease of LPX and proline for all pure volatiles except tetradecane compared to *F. udum*-treated plants indicates reduced infiltration of *Fusarium* in volatile-exposed plants. Antioxidant enzymes are essential in systemic resistance, serving as the initial defense mechanism that activates a series of responses to boost plant immunity against pathogens. They contribute by promoting the production of antimicrobial compounds and reinforcing cell walls (Appu et al. 2021). The observed increase in SOD and GPX activity in plants exposed to all volatiles except tetradecane indicates enhanced ROS scavenging, likely protecting the plants from oxidative damage and supporting their survival under stress. However, this heightened antioxidant response may not only reflect a protective mechanism against *Fusarium*-induced stress, it could also suggest that the volatiles themselves are causing oxidative stress. Some volatiles may be phytotoxic, triggering the plant’s antioxidant defenses as a direct response to their toxicity. To clarify whether the increased SOD and GPX activity is a response to *Fusarium* infection or a direct effect of the volatiles, a control experiment exposing plants to the volatiles alone, without *F. udum*, should be conducted to distinguish between these possibilities. Furthermore, a decrease in APX activity in volatile treated plants as compared to the infected control suggested the prioritization of glutathione-based ROS detoxification over ascorbate-dependent pathways. Complementarily, enhancement in levels of PPO indicates lignification, and might have led to improved resistance against wilt by producing quinones with antimicrobial properties. However, the evaluation of stress markers and antioxidant enzymes offered a mechanistic understanding of how these volatiles might contribute to higher resistance in pigeonpea plants against *Fusarium* wilt. Also, looking into the chitinase activity of infected plants in the presence of these VOCs could help to decipher their mode of action. Moreover, the *in planta* assays conducted in this study did not include a standard fungicide, such as carbendazim, mancozeb, antracol, etc., commonly reported in the literature to significantly reduce wilt incidence in pigeonpea (Gupta et al. 2014). This would have provided a useful reference point for assessing the relative efficacy of VOCs. While direct quantitative comparison is not possible due to differences in experimental setups, pathogen inoculum, and application doses, published data on fungicides can serve as a useful context for interpreting the biological relevance of the VOC-mediated protection observed here. Additionally, as the study was more focused on biological effects rather than absolute concentrations of the compounds, bacterial volatiles and synthetic analogs within plant tissues were not quantified. Techniques such as solid phase microextraction (SPME) or two-dimensional gas chromatography coupled to a time-of-flight mass spectrometer (TD-GC × GC-TOFMS) could be used in future experiments to better relate exposure levels of volatiles to plant responses (Schmidt et al. 2024). Moreover, VOCs are known to modulate rhizosphere microbial community composition and abundance, potentially influencing plant health. Sillo et al. (2024) evaluated soil quality in urban flowerbeds by linking microbial community composition with VOC emission profiles, and reported a correlation between tetradecane emission and the presence of five fungal genera. The antifungal activity of undecanal may also alter the fungal community structure of the plant microbiome. Further, Chou et al. (2023) reported that linalool at 100 µM promoted the growth of *Mortierella* but inhibited *Orbilia*, indicating that this monoterpene can differentially influence fungal taxa and shape rhizosphere microbial dynamics of sorghum. Considering these reports, future experiments in could include the collection of rhizospheric soil from VOC-exposed plants to elucidate how these volatiles shape the structure and function of microbial communities associated with the host.

## Conclusion

Due to the limitations of current farming practices, the goal of agriculture has shifted towards finding newer approaches that allow high crop productivity even under stress conditions without disturbing the environmental balance. The application of volatiles emitted by antagonist bacterial strains for mitigation of vascular wilt in pigeonpea whilst promoting plant growth proved successful under controlled conditions. The tailored list of bacterial volatiles emitted as a response to fungal volatiles enabled a targeted selection of volatiles with potential activity against *F. udum*. Moreover, the plant growth experiments validated the disease resistance capacity of some of the selected synthetic volatiles under controlled conditions. The use of volatiles in agricultural crops still has several limitations that need to be overcome before their widespread use. Their effectiveness under natural field conditions remains unverified. Additionally, since *F. udum* is a vascular pathogen, plant inoculation with volatiles would be more effective through root exposure. Therefore, a suitable method for delivering volatiles to the roots of pigeonpea needs to be developed first. Despite these limitations, our study offers a promising alternative to existing strategies that have been ineffective in controlling the wilt disease, while also paving the way for addressing other plant diseases and promoting ecological balance within the agricultural ecosystem.

## Supplementary Information

Below is the link to the electronic supplementary material.

Supplementary file1 Figure S1 includes principal component analysis plots and heatmaps corresponding to differential emission of volatiles by the five bacterial strains in response to continual exposure of fungal volatiles vs incubated alone. Table S1 contains list of volatiles emitted by bacterial strains in the absence of VOCs released by *F. udum*. (PDF 797 KB)

## Data Availability

All data generated or analysed during this study are included in this published article [and its supplementary information files]. The 16S rRNA gene sequences for the bacterial isolates used in this study are available at NCBI GenBank under accession numbers PP683115, PP683117, PP683118, PP683122 and OQ346092.

## Declarations

### GenBank

https://www.ncbi.nlm.nih.gov/genbank/
