# Supplementary material for: Volatile-mediated inhibition of Fusarium wilt in pigeonpea conferred by Bacillaceae members isolated from the host plant’s rhizosphere
Source: Plant Soil. 2025 Nov 26;527(2):1413–33. doi: 10.1007/s11104-025-08093-z (PMC13736443; doi:10.1007/s11104-025-08093-z)
Supplement: Supplementary file 1 [file 11104_2025_8093_MOESM1_ESM.pdf]

Volatile-mediated inhibition of *Fusarium* wilt in pigeonpea conferred by *Bacillaceae* members isolated from host plant rhizosphere

Rashi Tyagi[1], Sébastien Bruisson[2,3], Laure Weisskopf[2,3]*, Shilpi Sharma[1]*

[1]Department of Biochemical Engineering and Biotechnology, Indian Institute of Technology Delhi, New Delhi, 110016, India

[2]Department of Biology, University of Fribourg, Fribourg, 1700, Switzerland

[3]Food Research and Innovation Center, University of Fribourg, Fribourg, 1700, Switzerland

***Corresponding authors:**

Laure Weisskopf: laure.weisskopf@unifr.ch
Shilpi Sharma: shilpi@dbeb.iitd.ac.in

Plant and Soil Journal

Supplementary material

a)

(i)

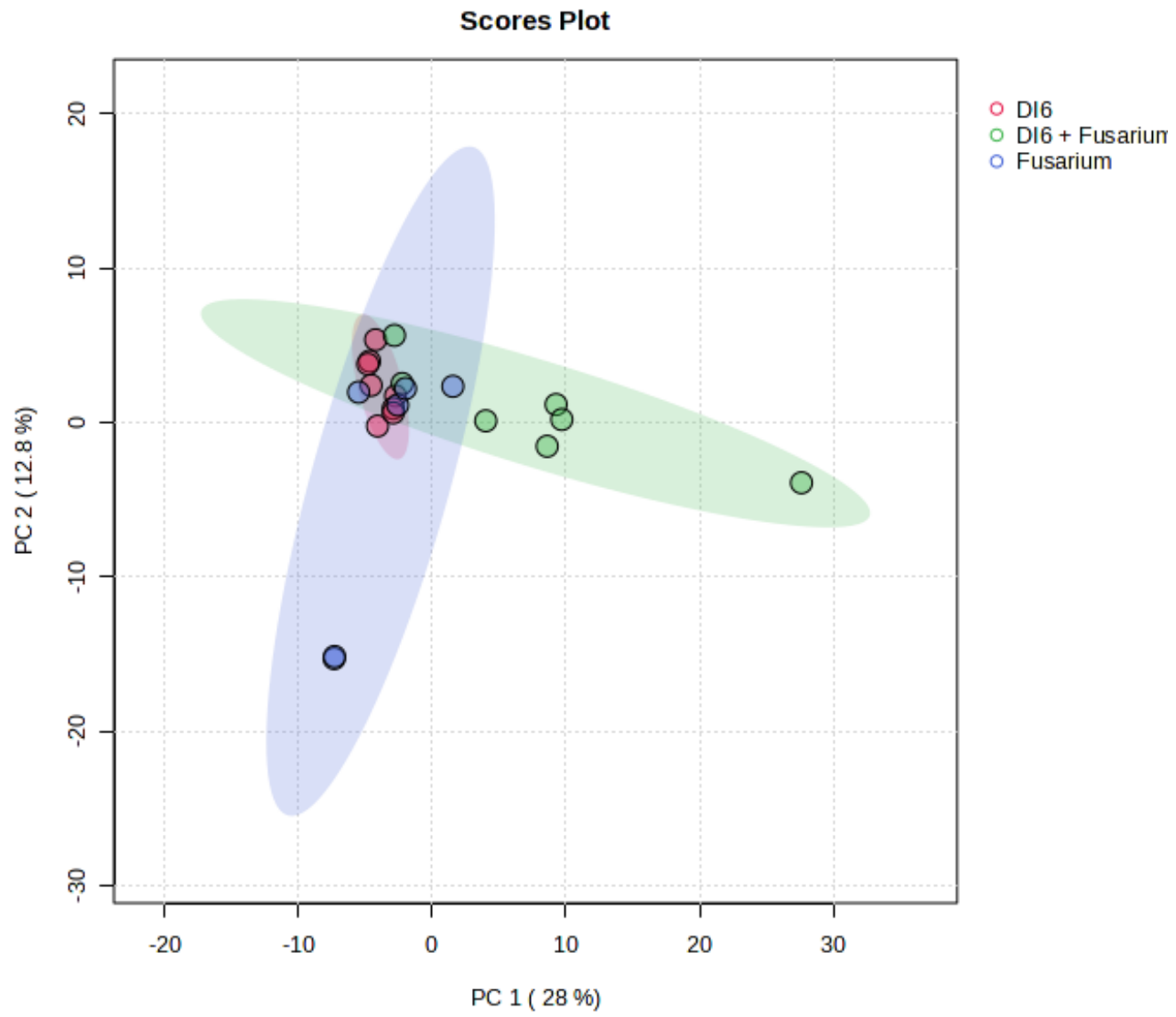

b)

(i)

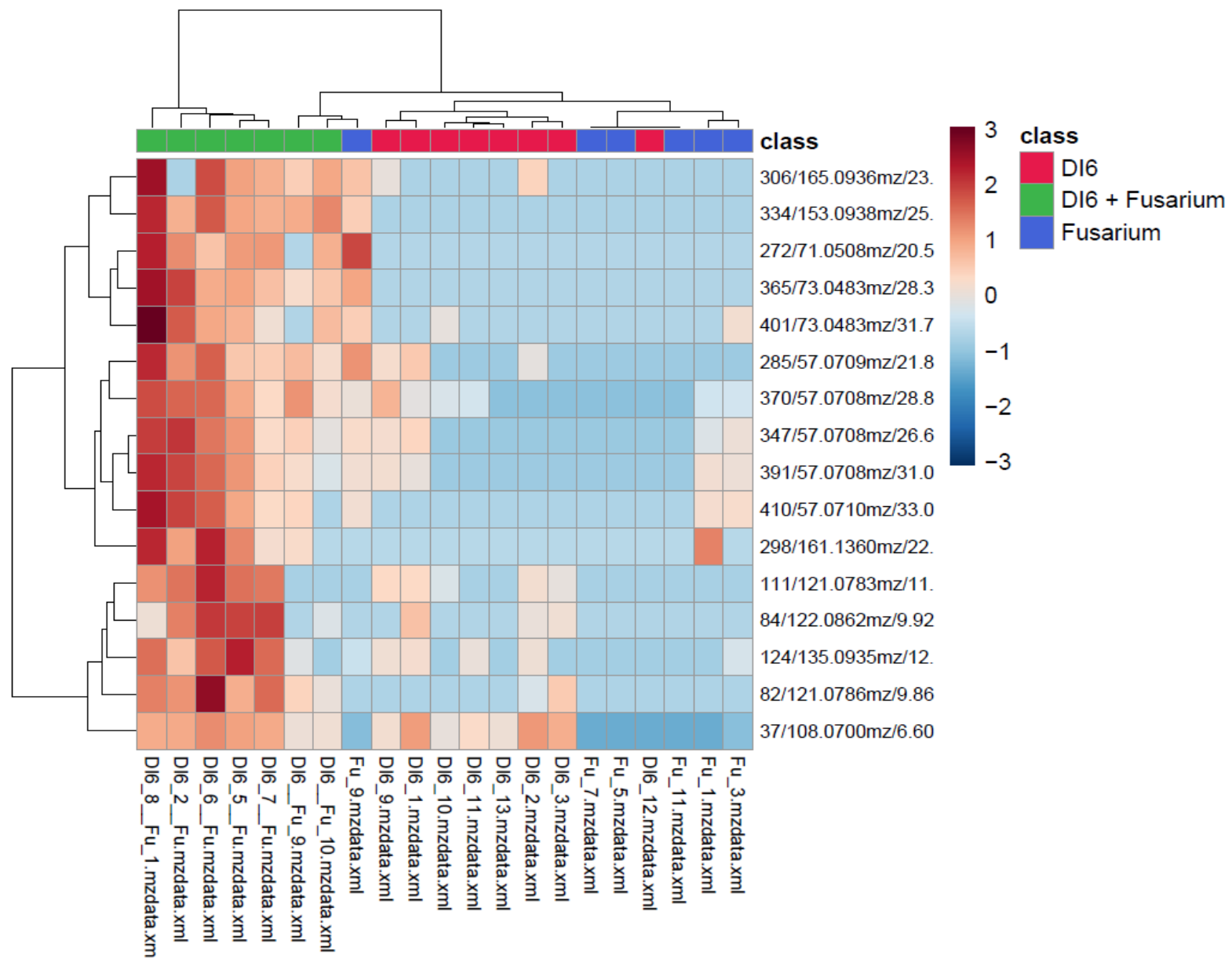

(ii)

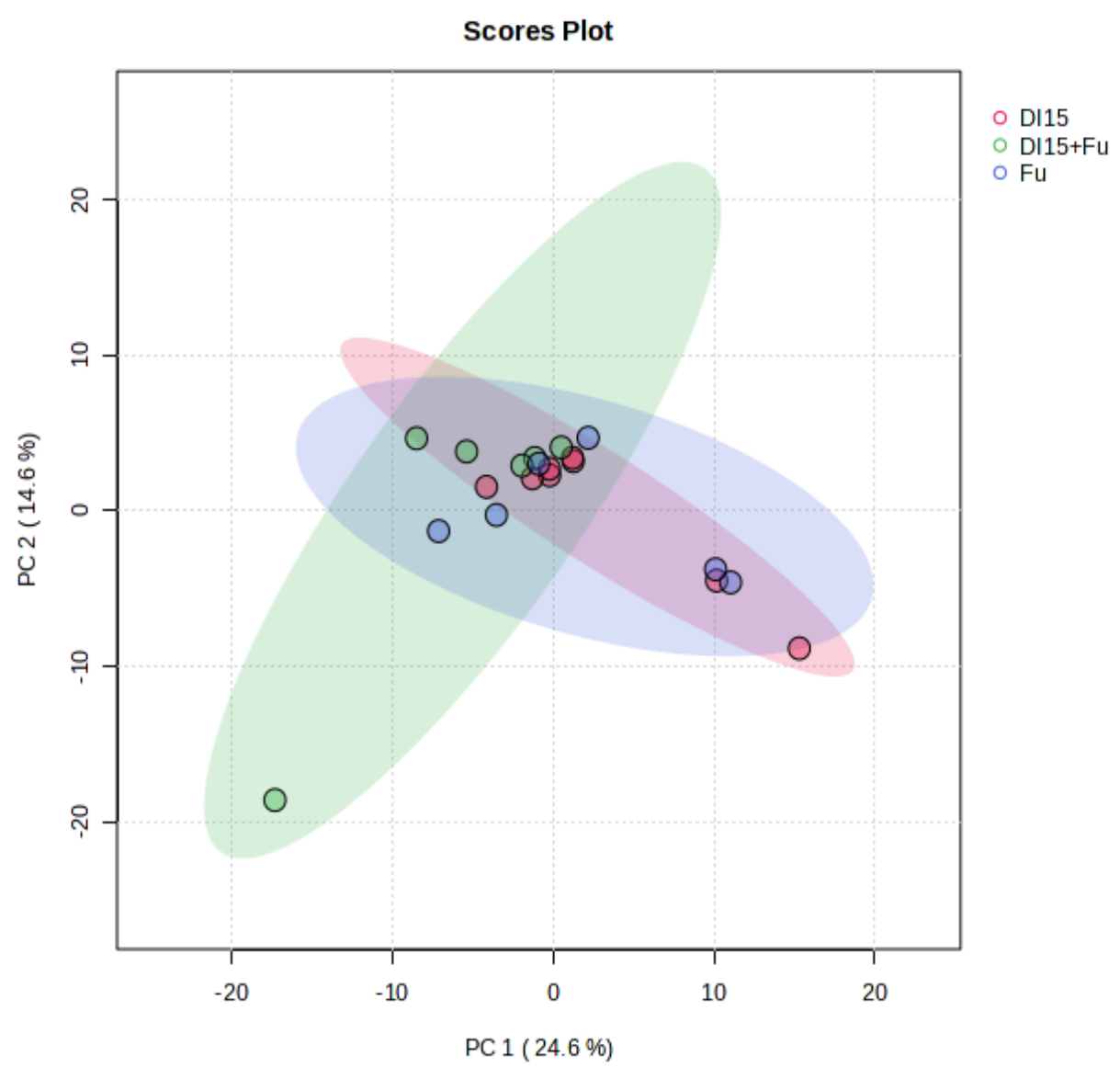

Scores Plot
DI15
DI15+Fu
Fu
PC 2 (14.6 %)
PC 1 (24.6 %)

(ii)

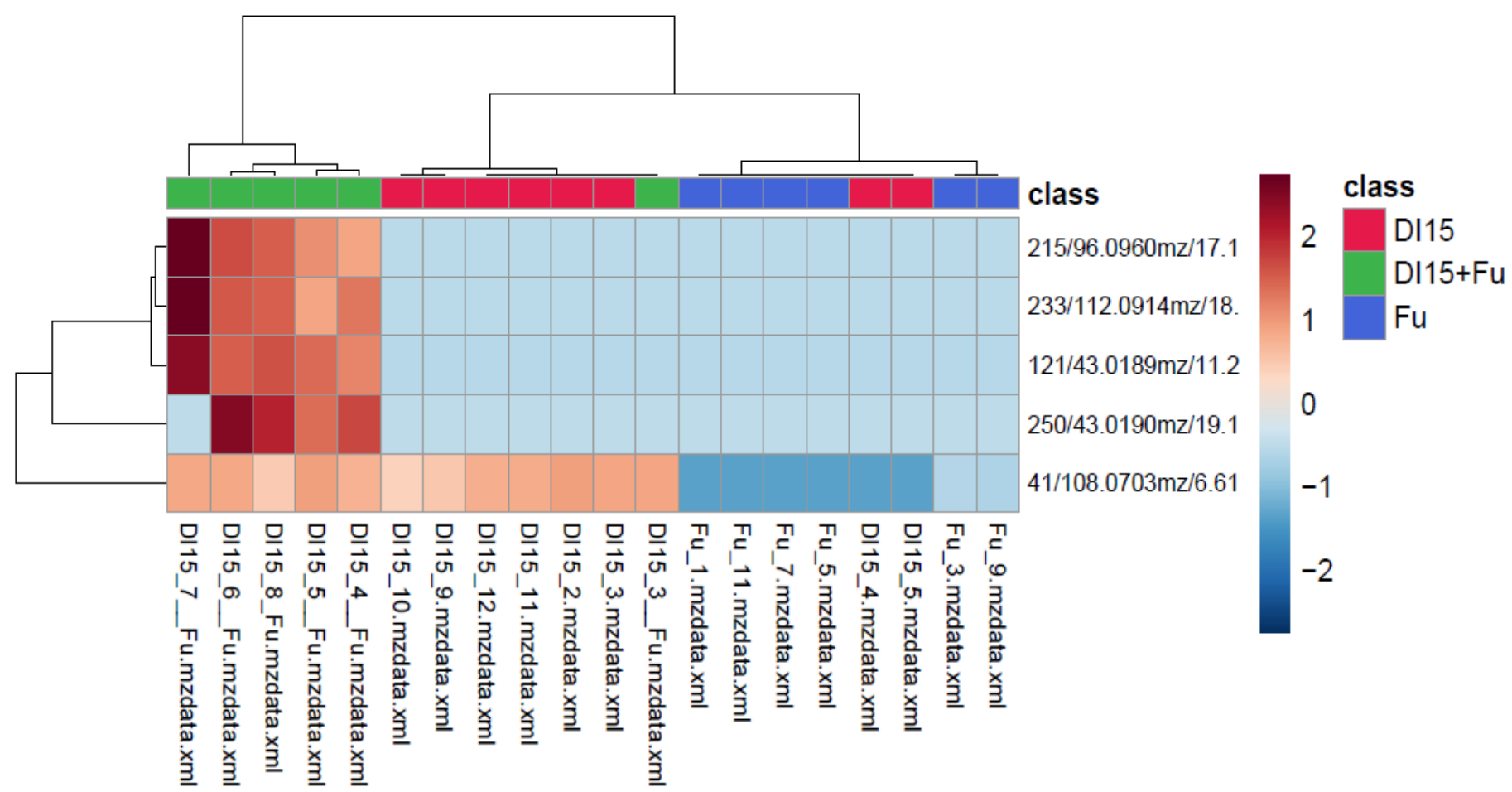

class
215/96.0960mz/17.1
233/112.0914mz/18.
121/43.0189mz/11.2
250/43.0190mz/19.1
41/108.0703mz/6.61
DI15_7__Fu.mzdata.xml
DI15_6__Fu.mzdata.xml
DI15_8_Fu.mzdata.xml
DI15_5__Fu.mzdata.xml
DI15_4__Fu.mzdata.xml
DI15_10.mzdata.xml
DI15_9.mzdata.xml
DI15_12.mzdata.xml
DI15_11.mzdata.xml
DI15_2.mzdata.xml
DI15_3.mzdata.xml
DI15_3__Fu.mzdata.xml
Fu_1.mzdata.xml
Fu_11.mzdata.xml
Fu_7.mzdata.xml
Fu_5.mzdata.xml
DI15_4.mzdata.xml
DI15_5.mzdata.xml
Fu_3.mzdata.xml
Fu_9.mzdata.xml
class
DI15
DI15+Fu
Fu

(iii)

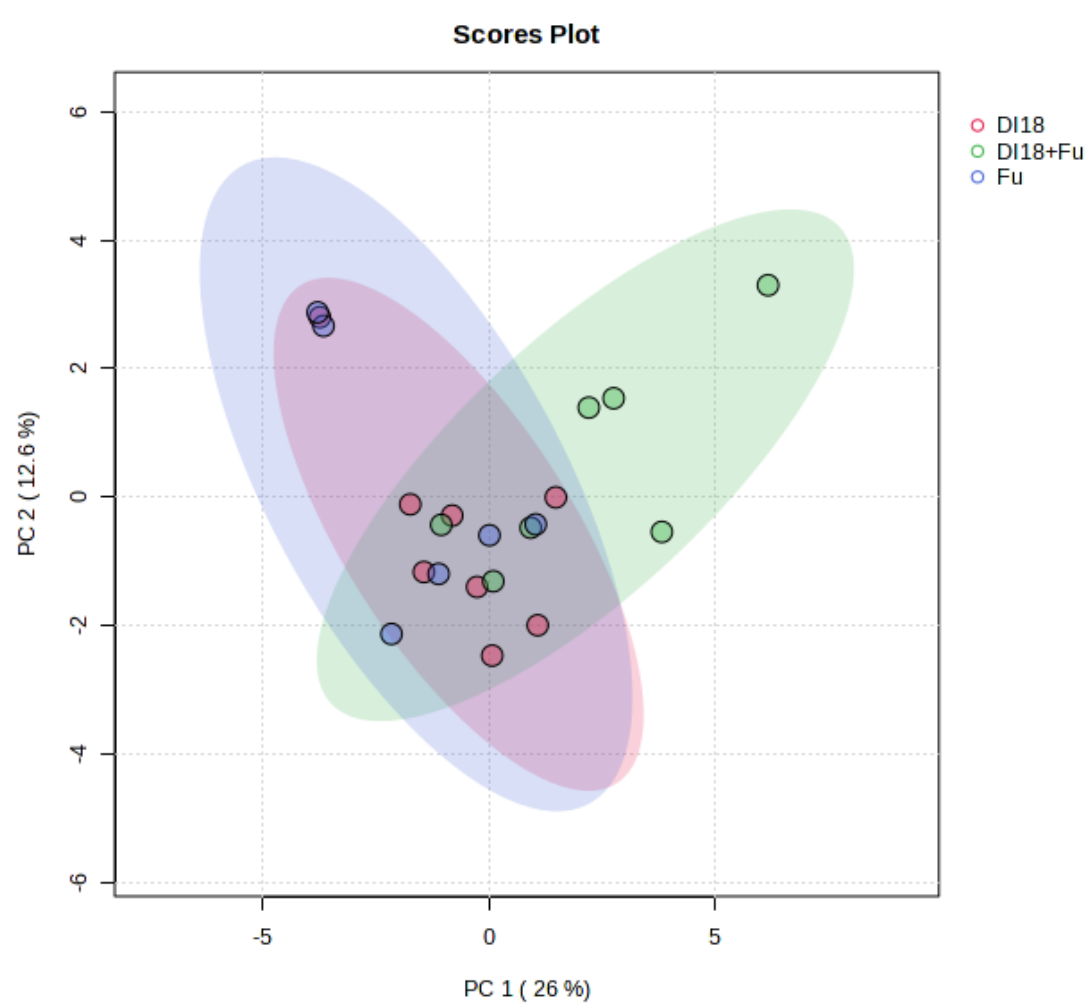

Scores Plot
DI18
DI18+Fu
Fu
PC 2 (12.6 %)
PC 1 (26 %)

(iii)

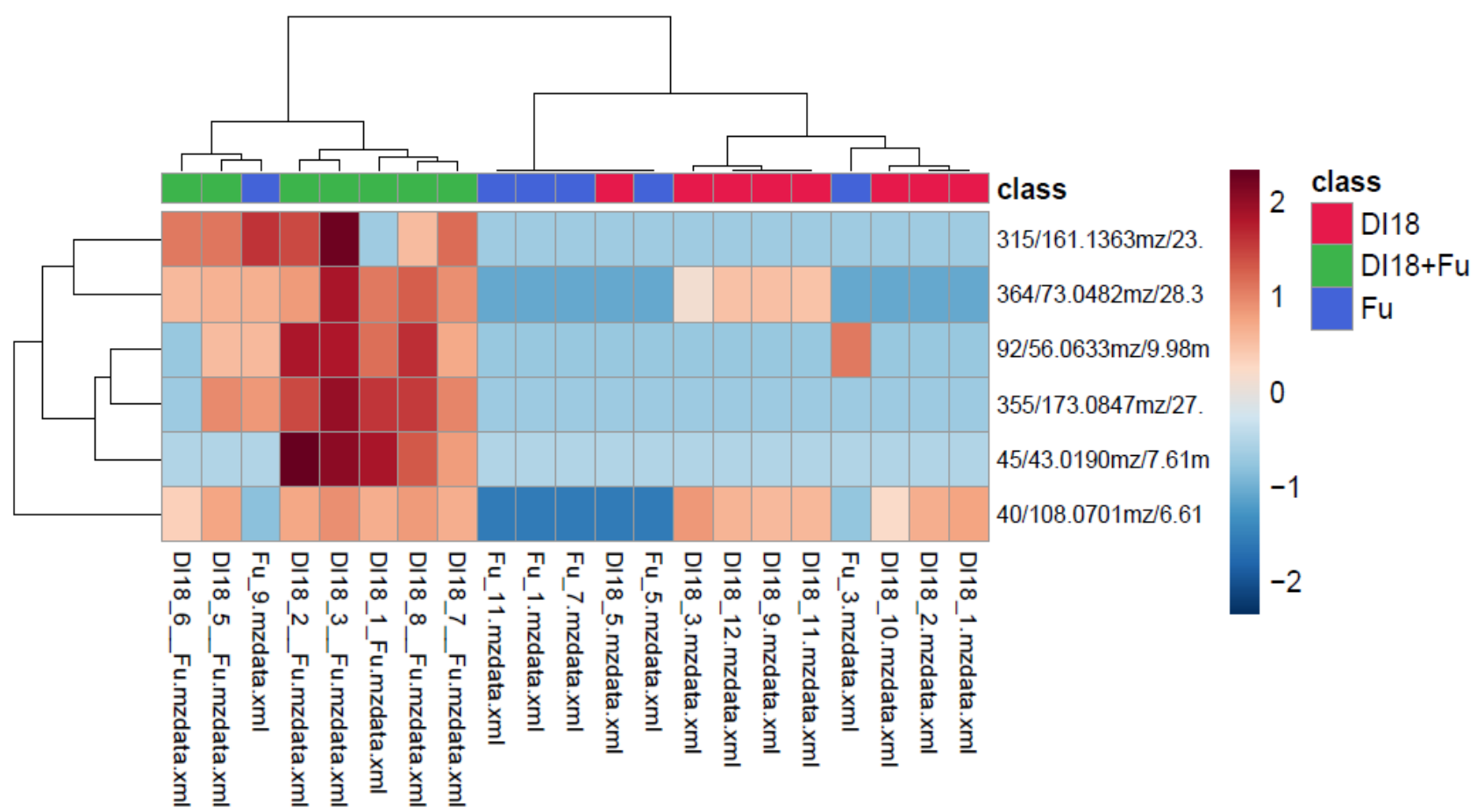

class
315/161.1363mz/23.
364/73.0482mz/28.3
92/56.0633mz/9.98m
355/173.0847mz/27.
45/43.0190mz/7.61m
40/108.0701mz/6.61
DI18_6__Fu.mzdata.xml
DI18_5__Fu.mzdata.xml
Fu_9.mzdata.xml
DI18_2__Fu.mzdata.xml
DI18_3__Fu.mzdata.xml
DI18_1_Fu.mzdata.xml
DI18_8__Fu.mzdata.xml
DI18_7__Fu.mzdata.xml
Fu_11.mzdata.xml
Fu_1.mzdata.xml
Fu_7.mzdata.xml
DI18_5.mzdata.xml
Fu_5.mzdata.xml
DI18_3.mzdata.xml
DI18_12.mzdata.xml
DI18_9.mzdata.xml
DI18_11.mzdata.xml
Fu_3.mzdata.xml
DI18_10.mzdata.xml
DI18_2.mzdata.xml
DI18_1.mzdata.xml
class
DI18
DI18+Fu
Fu

(iv)

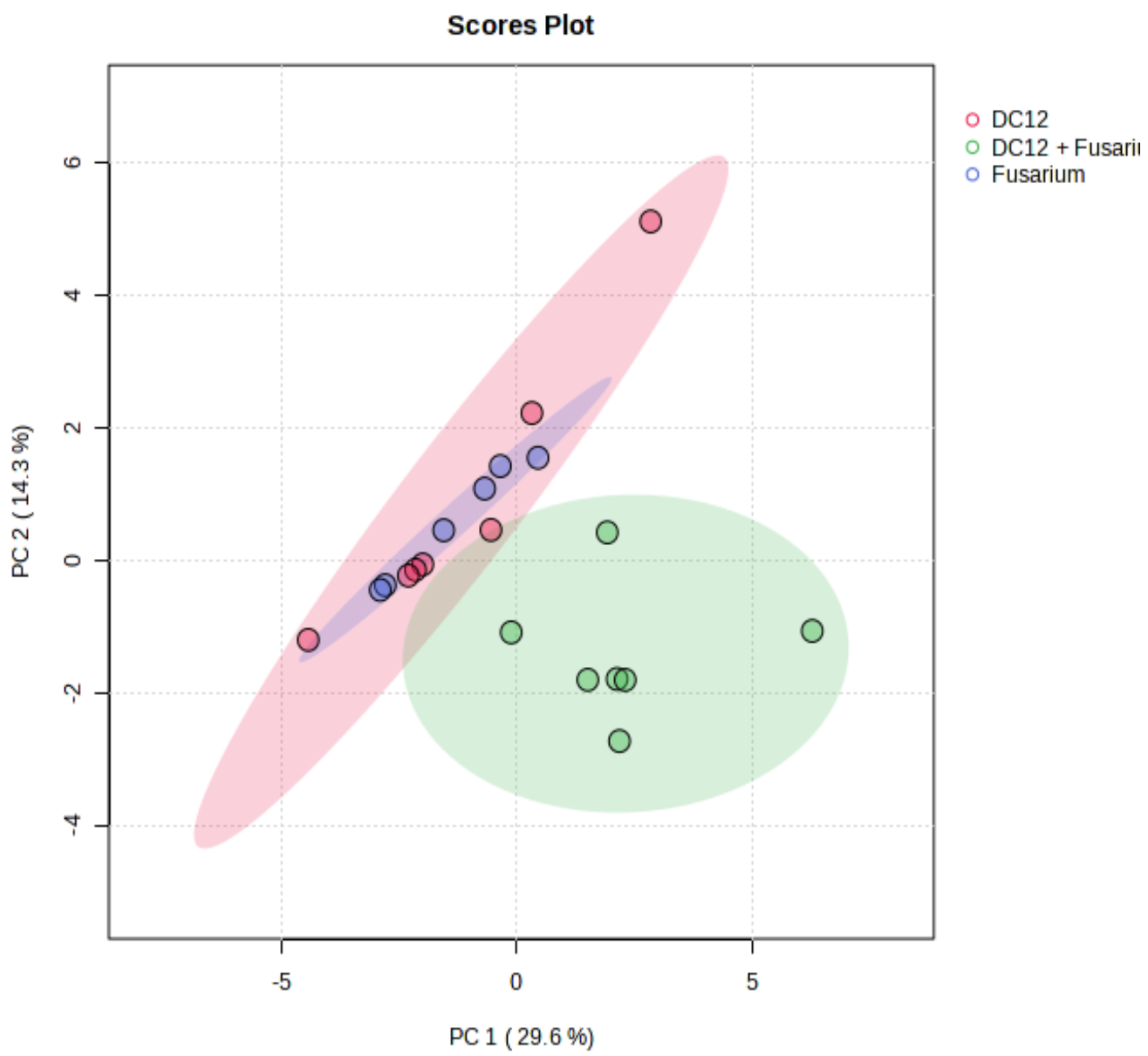

Scores Plot
DC12
DC12 + Fusari
Fusarium
PC 2 (14.3 %)
PC 1 (29.6 %)
-5
0
5
6
4
2
0
-2
-4

(iv)

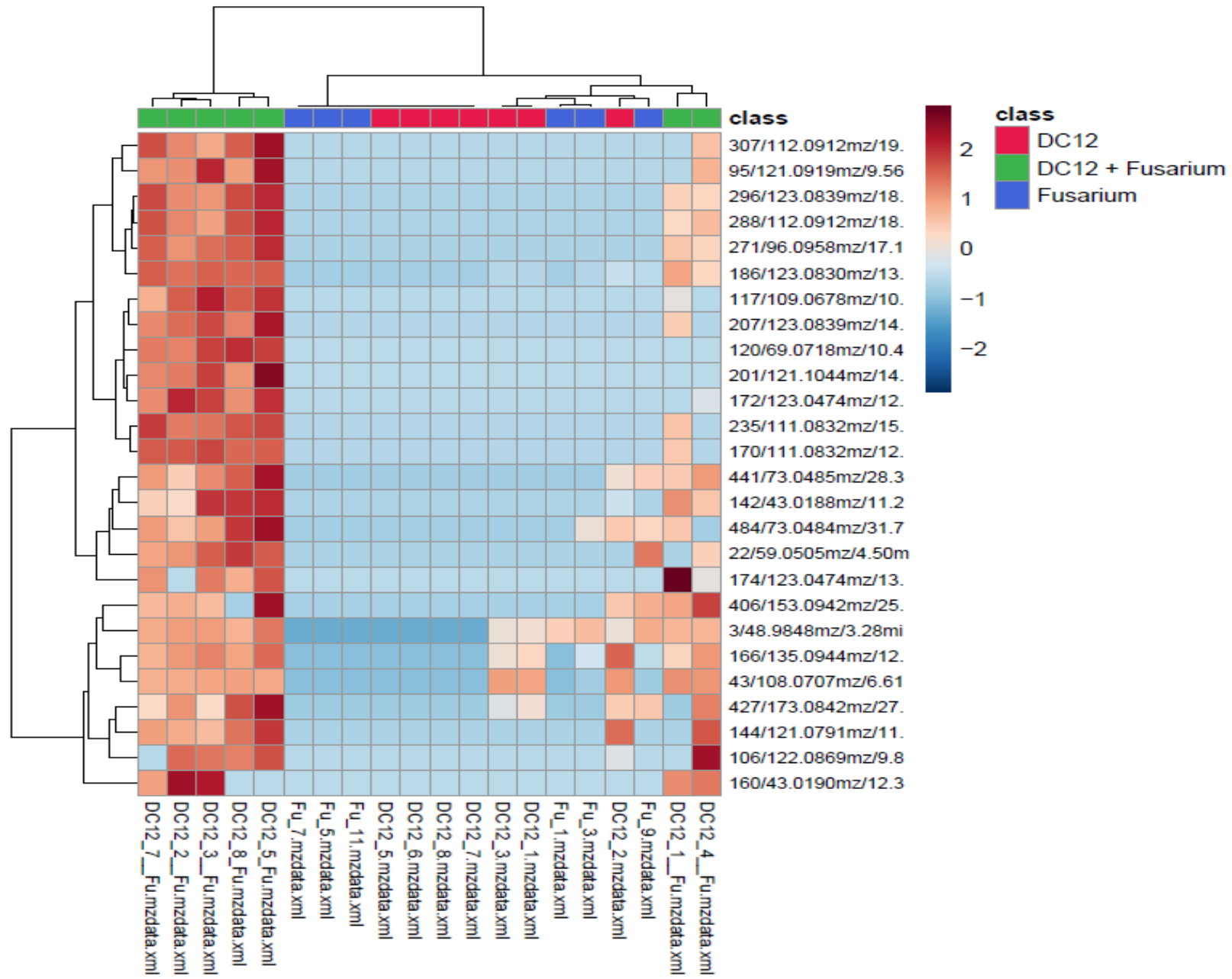

class
DC12
DC12 + Fusarium
Fusarium
2
1
0
−1
−2
307/112.0912mz/19.
95/121.0919mz/9.56
296/123.0839mz/18.
288/112.0912mz/18.
271/96.0958mz/17.1
186/123.0830mz/13.
117/109.0678mz/10.
207/123.0839mz/14.
120/69.0718mz/10.4
201/121.1044mz/14.
172/123.0474mz/12.
235/111.0832mz/15.
170/111.0832mz/12.
441/73.0485mz/28.3
142/43.0188mz/11.2
484/73.0484mz/31.7
22/59.0505mz/4.50m
174/123.0474mz/13.
406/153.0942mz/25.
3/48.9848mz/3.28mi
166/135.0944mz/12.
43/108.0707mz/6.61
427/173.0842mz/27.
144/121.0791mz/11.
106/122.0869mz/9.8
160/43.0190mz/12.3
DC12_7__Fu.mzdata.xml
DC12_2__Fu.mzdata.xml
DC12_3__Fu.mzdata.xml
DC12_8_Fu.mzdata.xml
DC12_5_Fu.mzdata.xml
Fu_7.mzdata.xml
Fu_5.mzdata.xml
Fu_11.mzdata.xml
DC12_5.mzdata.xml
DC12_6.mzdata.xml
DC12_8.mzdata.xml
DC12_7.mzdata.xml
DC12_3.mzdata.xml
DC12_1.mzdata.xml
Fu_1.mzdata.xml
Fu_3.mzdata.xml
DC12_2.mzdata.xml
Fu_9.mzdata.xml
DC12_1__Fu.mzdata.xml
DC12_4__Fu.mzdata.xml

(v)

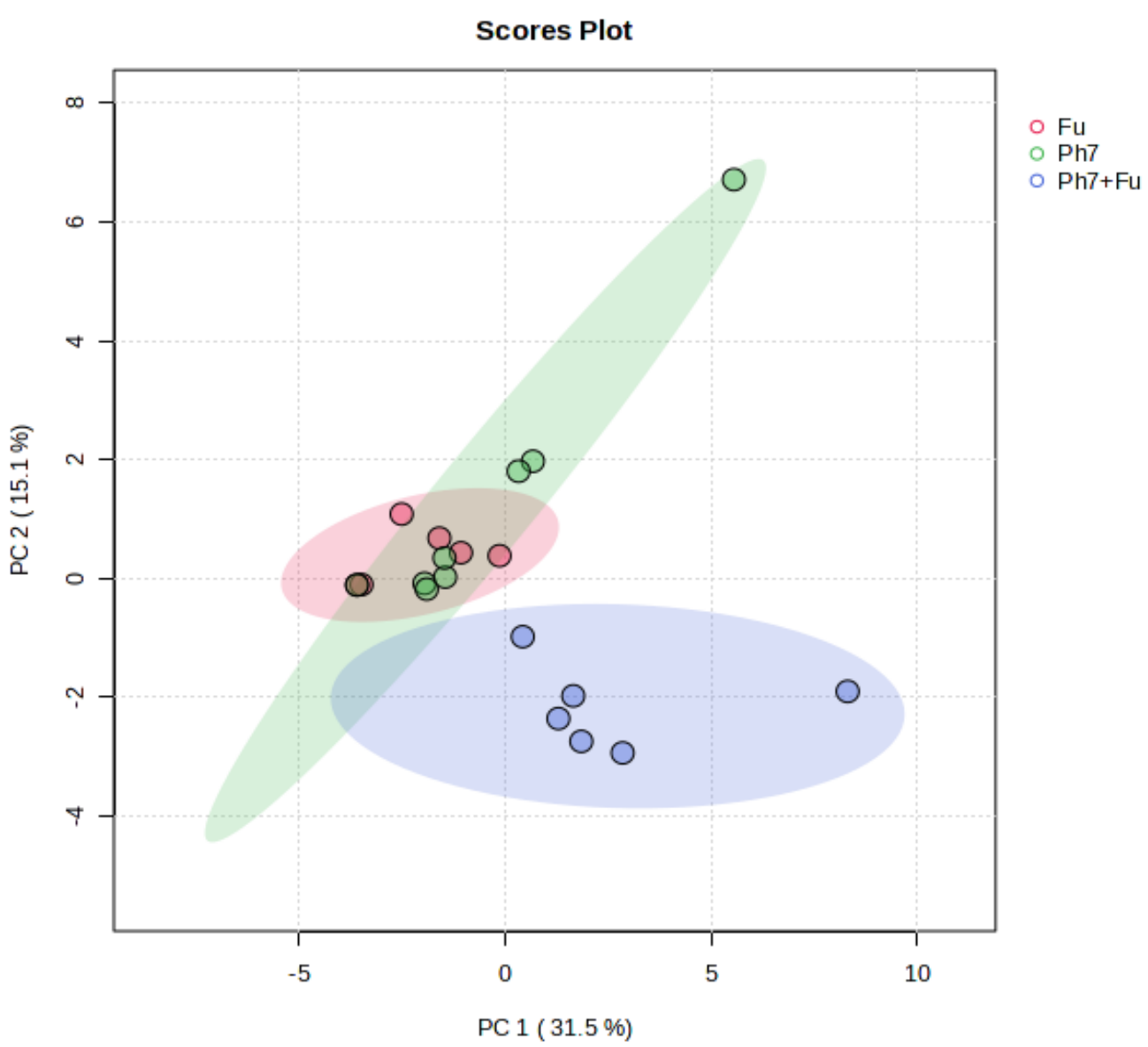

(v)

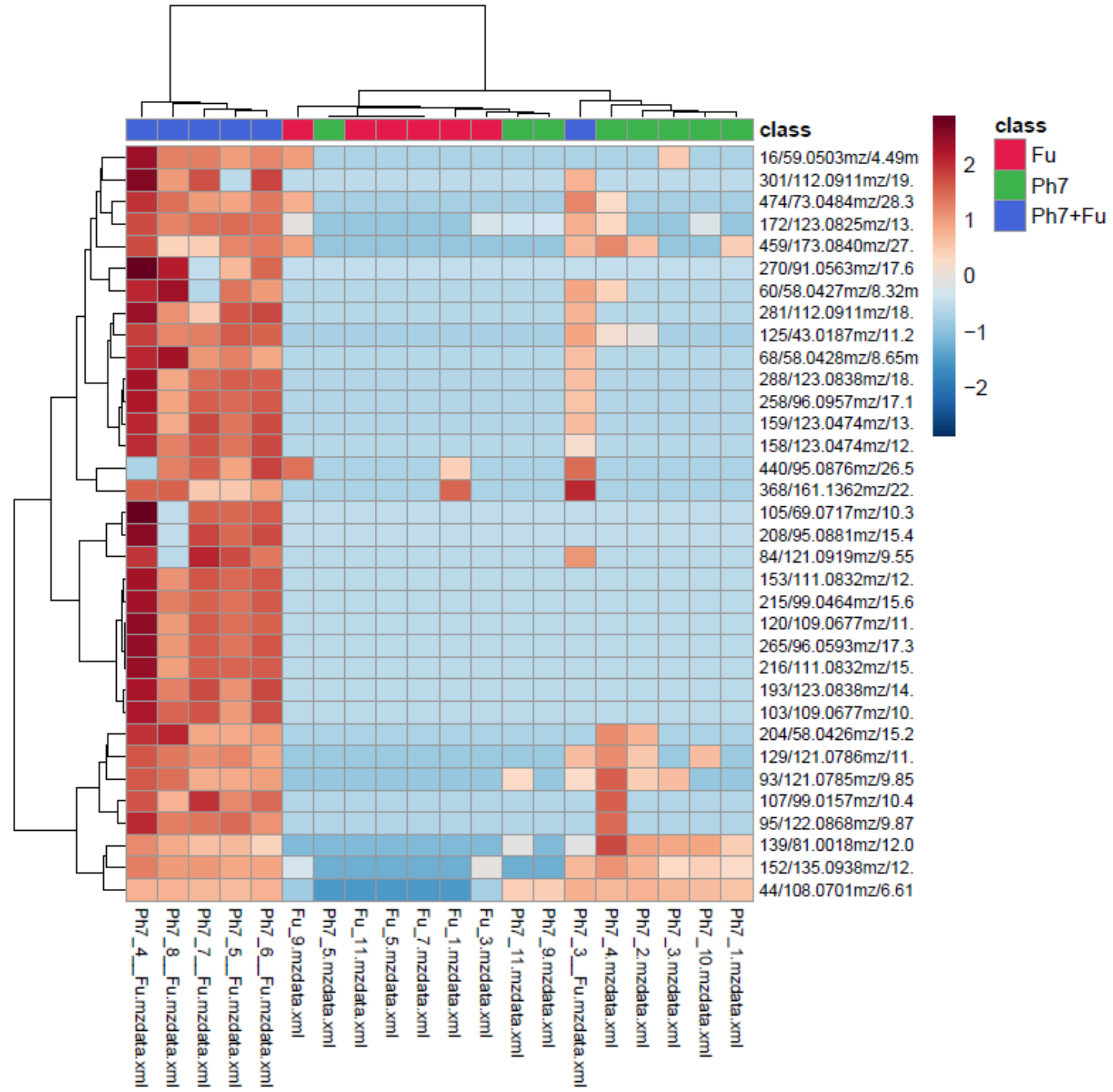

Figure S1. Differential emission of volatiles by the five bacterial strains in response to continual exposure of fungal volatiles vs incubated alone: a) Principal component analysis plots (variances in the bracket) and each dot represent one sample of volatilome; b) Heatmaps illustrating the hierarchical clustering of volatiles with significantly altered abundance across different groups. Each row corresponds to a volatile compound, while each column represents a sample. The colors indicate normalized intensity, and the dendrograms depict similarities.

Table S1. List of volatiles emitted by bacterial strains in the absence of VOCs emitted by *F. udum*

S. No.	Volatile organic compound	CAS number	Producer bacterial strains				
			DI6	DI15	DI18	DC12	Ph7
1	1-Tridecene	2437-56-1	✓		✓	✓	✓
2	Oxacyclododecan-2-one	1725-03-7	✓	✓		✓	✓
3	1-Undecanol	112-42-5	✓			✓	✓
4	2-Dodecanone	6175-49-1			✓	✓	✓
5	2-Methanesulfonylethanol	15205-66-0		✓	✓		✓
6	2-Naphthalenamine, N-phenyl-	135-88-6			✓	✓	✓
7	2-Nonanone	821-55-6			✓	✓	✓
8	2-Tridecanone	593-08-8			✓	✓	✓
9	Benzyl alcohol	100-51-6	✓	✓		✓	
10	Cyclohexane, nitro-	1122-60-7		✓	✓	✓	
11	Phenacyl thiocyanate	5399-30-4		✓		✓	✓
12	1,3,5-Benzenetriol	108-73-6				✓	✓
13	1,3-Butadiene, 2,3-dimethyl-	513-81-5		✓		✓	
14	1,5-Benzothiazepin-4(5H)-one, 2-methyl-	96680-77-2				✓	✓
15	2-Pentene, 3,4-dimethyl-, (Z)-	75-65-0		✓			✓
16	Benzene, (1-bromoethyl)-	585-71-7			✓	✓	
17	Benzene, 1,2,3-trimethyl-	526-73-8			✓	✓	
18	Dodecane	112-40-3				✓	✓
19	Flopropione	2295-58-1		✓		✓	
20	Heneicosane	629-94-7				✓	✓
21	Naphthalene	91-20-3				✓	✓
22	Pyridine, 4-(1,1-dimethylethyl)-	3978-81-2			✓		✓
23	Tetracosane	646-31-1			✓		✓
24	[1,1'-Biphenyl]-3-amine	2243-47-2		✓			✓
25	o-Xylene	95-47-6			✓	✓	
26	1(2H)-Naphthalenone, 3,4-dihydro-5,7-dimethyl-	13621-25-5					✓
27	1,2,3,4,5,6-Hexahydro-1,1,5,5-tetramethyl-2,4a-methanonaphthalen-7(4aH)-one	23747-14-0					✓
28	1,2-Benzenedicarboxylic acid, butyl cyclohexyl ester	84-64-0			✓		
29	1,4-Methanoazulen-7(1H)-one, octahydro-1,5,5,8a-tetramethyl-	65437-70-9				✓	
30	1-Butanamine	109-73-9					✓
31	1-Decanol	112-30-1	✓				
32	1-Hexanol, 2-ethyl-	104-76-7				✓	
33	1-Octadecanol	112-92-5					✓
34	1-Propanol, 3-(methylthio)-	505-10-2			✓		
35	1-Tetradecene	629-59-4					✓
36	1-Undecene	821-95-4			✓		
37	11-Hexadecen-1-ol, (Z)-	56683-54-6	✓				
38	1H-Pyrrole, 3-ethyl-2,4-dimethyl-	517-22-6			✓		
39	2(3H)-Furanone, dihydro-5-propyl-	105-21-5		✓			

40	2,3,6-Trichlorobenzaldehyde	4659-47-6				✓	
41	2,4,6-Trihydroxybenzoic acid	83-30-7			✓		
42	2-Aminopyridine	504-29-0				✓	
43	2-Furanmethanol	98-00-0	✓				
44	2-Hexanone, 5-methyl-	110-12-3					✓
45	2-Isobutylthiazole	18640-74-9			✓		
46	2-Pentene, 2,4-dimethyl-	625-65-0				✓	
47	2-Propanol, 2-methyl-	75-65-0		✓			
48	2-Pyrimidinamine, 4,6-dimethyl-				✓		
49	3-Pentanol, 2-methyl-	767-15-7					✓
50	4-Terpinenyl acetate	09-04-4821					✓
51	5-Chlorovaleric acid	1119-46-6					✓
52	9,10-Anthracenedione	84-65-1	✓				
53	9H-Fluorene, 9-bromo-	1940-57-4					✓
54	Acetamide, N-[3-(trifluoromethyl)phenyl]-	351-36-0					✓
55	Acetoin	513-86-0				✓	
56	Adenine	73-24-5			✓		
57	Aminopyrine	58-15-1				✓	
58	Anthracene	120-12-7					✓
59	Azelaic acid	123-99-9					✓
60	Benzaldehyde, 2,3,4-trimethoxy-	2103-57-3					✓
61	Benzaldehyde, 2,4,5-trimethoxy-	4460-86-0					✓
62	Benzene, 1,2,4-trimethyl-	95-63-6					✓
63	Benzene, 1-ethyl-3-methyl-	620-14-4					✓
64	Benzothiazole, 2-methyl-	120-75-2			✓		
65	Brucine	357-57-3					✓
66	Cholest-5-en-3-ol (3ß)-, propanoate	633-31-8					✓
67	Cyclohexane, 1,2-dimethyl-(cis/trans)	2207-01-4/6876-23-9				✓	
68	Cyclohexane, ethyl-	1678-91-7					✓
69	Cyclohexanone, 2-nitro-	4883-67-4				✓	
70	Cyclohexanone, 3,5-dimethyl-	2320-30-1				✓	
71	Decane	124-18-5				✓	
72	Dodecanal	112-40-3				✓	
73	Equilin	474-86-2					✓
74	Ergoline-8-methanol, 8,9-didehydro-6-methyl-	548-43-6		✓			
75	Ethanedione, diphenyl-	134-81-6			✓		
76	Ethanol, 2-(2,4-dichlorophenoxy)-	120-67-2					✓
77	Ethyl N,N-dimethyloxamate	16703-52-9		✓			
78	Heptadecane	629-78-7					✓
79	Hexanoic acid, 2-hexenyl ester, (E)-	53398-86-0			✓		
80	Lysergamide	478-94-4	✓				
81	Methanone, [4-(dimethylamino)phenyl]phenyl-	530-44-9					✓
82	Naphthalene, 1-(1-methylethyl)-	6158-45-8					✓

83	Naphthalene, 1-chloro-	90-13-1				✓	
84	Octane, 3,5-dimethyl-	15869-93-9					✓
85	Pentane, 2,2,4-trimethyl-	540-84-1				✓	
86	Phenylethyl Alcohol	60-12-8		✓			
87	Physostigmine	57-47-6					✓
88	Prednisolone Acetate	52-21-1			✓		
89	Propane, 2-methoxy-2-methyl-	1634-04-4					✓
90	Propanoic acid, 3-chloro-	107-94-8				✓	
91	Silane, chloro(1,1-dimethylethyl)diphenyl-	58479-61-1					✓
92	Silane, trichloro(chloromethyl)-	1558-25-4	✓				
93	Stannane, fluorotriisobutyl-	7304-48-5	✓				
94	Succinic anhydride	108-30-5			✓		
95	Tridecanoic acid, methyl ester	1731-88-0					✓
96	Undecanoic acid, 2-methyl-	24323-25-9					✓
97	n-Hexyl salicylate	6259-76-3					✓
98	n-Tridecan-1-ol	112-70-9	✓				
99	α-Chloroethyltrimethylsilane	7787-87-3				✓	

Note: ✓represents the emission of volatile by strain/s listed in that column.
